# Fast Acoustic Steering via Tilting Electromechanical Reflectors (FASTER): A Novel Method for High Volume Rate 3-D Ultrasound Imaging

**DOI:** 10.1109/TUFFC.2020.3020871

**Published:** 2021-02-25

**Authors:** Zhijie Dong, Shuangliang Li, Matthew R. Lowerison, Jason Pan, Jun Zou, Pengfei Song

**Affiliations:** Beckman Institute, University of Illinois at Urbana–Champaign, Urbana, IL 61801 USA, and also with the Department of Electrical and Computer Engineering, University of Illinois at Urbana–Champaign, Urbana, IL 61801 USA; Department of Electrical and Computer Engineering, Texas A&M University, College Station, TX 77843 USA.; Beckman Institute, University of Illinois at Urbana–Champaign, Urbana, IL 61801 USA, and also with the Department of Electrical and Computer Engineering, University of Illinois at Urbana–Champaign, Urbana, IL 61801 USA; Department of Electrical and Computer Engineering, University of Illinois at Urbana–Champaign, Urbana, IL 61801 USA.; Department of Electrical and Computer Engineering, Texas A&M University, College Station, TX 77843 USA.; Beckman Institute, University of Illinois at Urbana–Champaign, Urbana, IL 61801 USA, and also with the Department of Electrical and Computer Engineering, University of Illinois at Urbana–Champaign, Urbana, IL 61801 USA

**Keywords:** 3-D ultrasound, high volume rate, plane wave imaging, water-immersible microfabricated mirror

## Abstract

The 3-D ultrasound imaging is essential for a wide range of clinical applications in diagnostic and interventional cardiology, radiology, and obstetrics for prenatal imaging. 3-D ultrasound imaging is also pivotal for advancing technical developments of emerging imaging technologies, such as elastography, blood flow imaging, functional ultrasound (fUS), and super-resolution microvessel imaging. At present, however, existing 3-D ultrasound imaging methods suffer from low imaging volume rate, suboptimal imaging quality, and high costs associated with 2-D ultrasound transducers. Here, we report a novel 3-D ultrasound imaging technique, fast acoustic steering via tilting electromechanical reflectors (FASTER), which provides both high imaging quality and fast imaging speed while at low cost. Capitalizing upon unique water immersible and fast-tilting microfabricated mirror to scan ultrafast plane waves in the elevational direction, FASTER is capable of high volume rate, large field-of-view (FOV) 3-D imaging with conventional 1-D transducers. In this article, we introduce the fundamental concepts of FASTER and present a series of calibration and validation studies for FASTER 3-D imaging. In a wire phantom and tissue-mimicking phantom study, we demonstrated that FASTER was capable of providing spatially accurate 3-D images with a 500-Hz imaging volume rate and an imaging FOV with a range of 48° (20 mm at 25-mm depth) in the elevational direction. We also showed that FASTER had comparable imaging quality with conventional mechanical translation-based 3-D imaging. The principles and results presented in this study establish the technical foundation for the new paradigm of high volume rate 3-D ultrasound imaging based on ultrafast plane waves and fast-tilting, water-immersible microfabricated mirrors.

## I. Introduction

Ultrasound has become the most commonly used clinical imaging modality due to its safety, low cost, and portability. Its high imaging frame rate allows operators to perform clinical diagnosis in real time [1], enabling rapid screening and image-guided interventional procedures. However, conventional ultrasound can only provide a 2-D image for 3-D tissue structures. This leads to a high degree of operator dependence and uncertainty in image-guided procedures because radiological assessment, targeting, and image quantifications are dependent on transducer placement and patient positioning. Furthermore, ultrasound operators must mentally integrate 3-D anatomy during the scan, a skill that takes a substantial amount of training and is associated with poor interobserver reproducibility [2].

With the capabilities of volumetric data acquisition, visualization, and quantification, 3-D ultrasound can mitigate the issues of operator dependence and uncertainties of imaging guidance associated with 2-D imaging. These features facilitate more reliable radiological evaluations and interpretations as well as more robust interventional planning with ultrasound. In addition, 3-D ultrasound has the potential of offering more accurate quantitative measurements with improved repeatability, accuracy, and reproducibility over 2-D imaging [3].

Although it has much to offer over its 2-D counterpart, current 3-D ultrasound imaging techniques are largely limited in providing a clinically useful dynamic and accurate imaging information due to the slow volumetric sampling rate and suboptimal imaging quality than 2-D imaging. Current implementations of 3-D ultrasound can be largely categorized into three groups: mechanical scanning (i.e., wobbler or sweeper), sensor-tracking-based scanning, and 3-D imaging with 2-D ultrasound transducers [4]–[6]. Mechanical scanning involves an external assembly to translate, rotate, tilt, or sweep a 1-D ultrasound transducer to cover a 3-D volume. This group of techniques is relatively simple to implement and computationally low cost to operate although the imaging volume rate can be limited (0.2–3 Hz [6]) due to the need for mechanically moving the transducer. Also, although the in-plane (i.e., azimuthal) imaging resolution is similar to 2-D imaging for the mechanical translation-based 3-D imaging, the out-of-plane (i.e., elevational) imaging resolution is significantly worse due to the lack of transmit and receive focusing in the elevational direction. The second group of methods is a free-form version of the mechanical scanning, which is based on sensor-tracking-enabled freehand 3-D acquisition [7], [8]. This class of methods uses either an articulated mechanical arm, a magnetic or optical tracking sensor [9]–[12], or ultrasound-based speckle decorrelation [12], [13] to provide transducer positioning information, which enables individual 2-D imaging frames to be assembled into a 3-D volume with arbitrary dimensions and shapes. Since the sensor-tracking-based approaches only involve 1-D ultrasound transducers and low-cost sensors, they offer a distinct advantage for easy integration into clinical practice with existing clinical ultrasound systems. However, similar to the mechanical scanning technique, this group of methods suffers from low 3-D imaging volume rate and suboptimal elevational imaging resolution. Also, the accuracy and resolution of the sensor may be affected by interferences or obstacles, which leads to the misregistration of 2-D images and deteriorated 3-D imaging quality [4]. The third group of methods for 3-D ultrasound imaging is based on 2-D ultrasound transducers [14], which provides a direct volumetric insonification of the targeted medium for 3-D imaging. Without the need for mechanically moving the ultrasound transducer, this class of techniques offers the best volume sampling rate, which is on the order of 10^0^–10^1^ Hz [4]. Some low element-count transducers can achieve a high volume rate when combined with a high channel-count ultrasound system (e.g., 200–2000 Hz for a 32 × 32 2-D array with four Vantage systems and 1024 channels [15]). Also, 2-D transducers provide a more isotropic imaging resolution in both azimuthal and elevational dimensions than the mechanical-translation-based methods although the in-plane imaging resolution can be worse than conventional 2-D imaging. However, a major limiting factor that prevents this class of techniques from being widely used in clinical practice is the cost and complexities associated with the 2-D transducers: the number of transducer elements is typically on the order of 10^3^–10^4^, which needs a system with high channel count to operate, and is challenging to fabricate and computationally costly to conduct beamforming and postprocessing. Although some of these challenges can be mitigated by instrumentation and computational innovations, such as multiplexing, microbeam-forming [16]–[18], parallel beamforming, and novel arrays, such as sparse arrays [19]–[21] and row–column-addressing (RCA) arrays [22]–[24], a viable solution that provides both high imaging quality and high 3-D imaging volume rate remains elusive until today.

Inspired by the recent development of the MEMS-based fast scanning techniques in photoacoustic microscopy (PAM), here, we present a novel 3-D ultrasound imaging method that combines conventional 1-D ultrasound transducers with water-immersible and fast-tilting microfabricated scanning mirrors. In PAM, a similar challenge of low scanning rate related to the translation of mechanical stages was addressed by the use of a water-immersible MEMS scanning mirror, which provided a B-scan imaging speed up to 400 Hz [25] (about 400 times faster than the second-generation optical resolution PAM system [26]). In ultrasound, the idea of using a mirror or a reflector to redirect and extend the imaging field-of-view (FOV) was previously described in forward-viewing imaging catheters [27], where a mirror was used in combination with a rotating ultrasound transducer to achieve forward-viewing 2-D imaging. Motivated by the capabilities of the water-immersible microfabricated mirror and the feasibility of performing ultrasound imaging with a mirror reflector, here, we propose to combine ultrasound with the microfabricated mirror and leverage the ultrafast imaging frame rate of plane waves to achieve high volume rate 3-D imaging. Our technique, called fast acoustic steering via tilting electromechanical reflectors (FASTER), distributes the redundancy of the ultrafast imaging frame rate of plane waves in the elevational direction by using a microfabricated mirror that rapidly sweeps the plane waves to form a wedge-shaped 3-D FOV. Similar to a wobbler transducer, FASTER enjoys high azimuthal imaging quality. However, FASTER has a much higher volume imaging rate over the wobblers because FASTER sweeps the ultrasound beam instead of the ultrasound transducer. In addition, FASTER outperforms conventional 2-D transducers in imaging volume rate by approximately an order of magnitude, is associated with a much lower cost to fabricate and operate, and can be conveniently adopted by existing commercial systems with limited channel count.

The rest of this article is structured as follows. We first describe the principles of FASTER, followed by descriptions of the design of the microfabricated mirror, the experimental setup for the mirror-based beam reflection study, the acoustic calibration study, and, finally, the phantom imaging study. The results of corresponding studies will be presented in Section III. We finalize this article with discussion and conclusions.

## II. Materials and Methods

### A. Principles of FASTER

FASTER generates 3-D images with a high imaging volume rate through the steering of ultrafast plane waves with a fast-tilting reflector. The central idea of FASTER is that since ultrafast plane wave imaging achieves tens of thousands of Hertz of frame rate, one can distribute the imaging planes at different elevational directions to realize 3-D imaging instead of imaging the same location repeatedly.

Revisiting the theoretical pulse repetition frequency (PRF) of a pulse–echo plane wave imaging system

(1) $$\text{PRF}=\frac{1}{t}=\frac{c}{2d}$$

where *t* is the round-trip ultrasound traveling time (i.e., pulse repetition interval), *c* is the speed of sound in tissue, and *d* is the imaging depth. When spatial angular compounding imaging [28] is used, PRF is reduced to effective PRF_*e*_

(2) $${\text{PRF}}_{e}=\frac{\text{PRF}}{n_{a}}$$

where *n*_*a*_ is the number of compounding angles. When distributing the imaging planes along the elevational dimension via a fast-tilting microfabricated mirror, with a tilting frequency of *F*_*m*_, the tilting angle (*θ*_*n*_) of the microfabricated mirror with a sinusoidal driving signal corresponding to the *n*th imaging plane is given by

(3) $$\theta_{n}=A\;\text{sin}\left(2\pi \;F_{m}t_{n}+\phi \right)+\gamma \;=A\;\text{sin}\left(2\pi \;F_{m}\frac{n}{{\text{PRF}}_{e}}+\phi \right)+\gamma$$

where *A* is the half-side range of the tilting angle of the microfabricated mirror, *t*_*n*_ is the time to sample the *n*th imaging plane (*n* = 1, 2, … *, N*_*p*_ and $n\in {\mathbb{Z}}^{+}$), ${\mathbb{Z}}^{+}$ denotes positive integer number, *ϕ* is the initial phase of the microfabricated mirror, and *γ* is the tilting angle offset of the mirror. Since the incident angle is equal to the reflection angle of an acoustic wave, the scanning angle (*α*_*n*_) of the microfabricated mirror is twice of the tilting angle (e.g., scanning angle is changed by 90° when the mirror is tilted by 45°)

(4) $$\alpha_{n}=2\theta_{n}=2A\;\text{sin}\left(2\pi \;F_{m}\frac{n}{{\text{PRF}}_{e}}+\phi \right)+2\gamma .$$

**Fig. 1.**
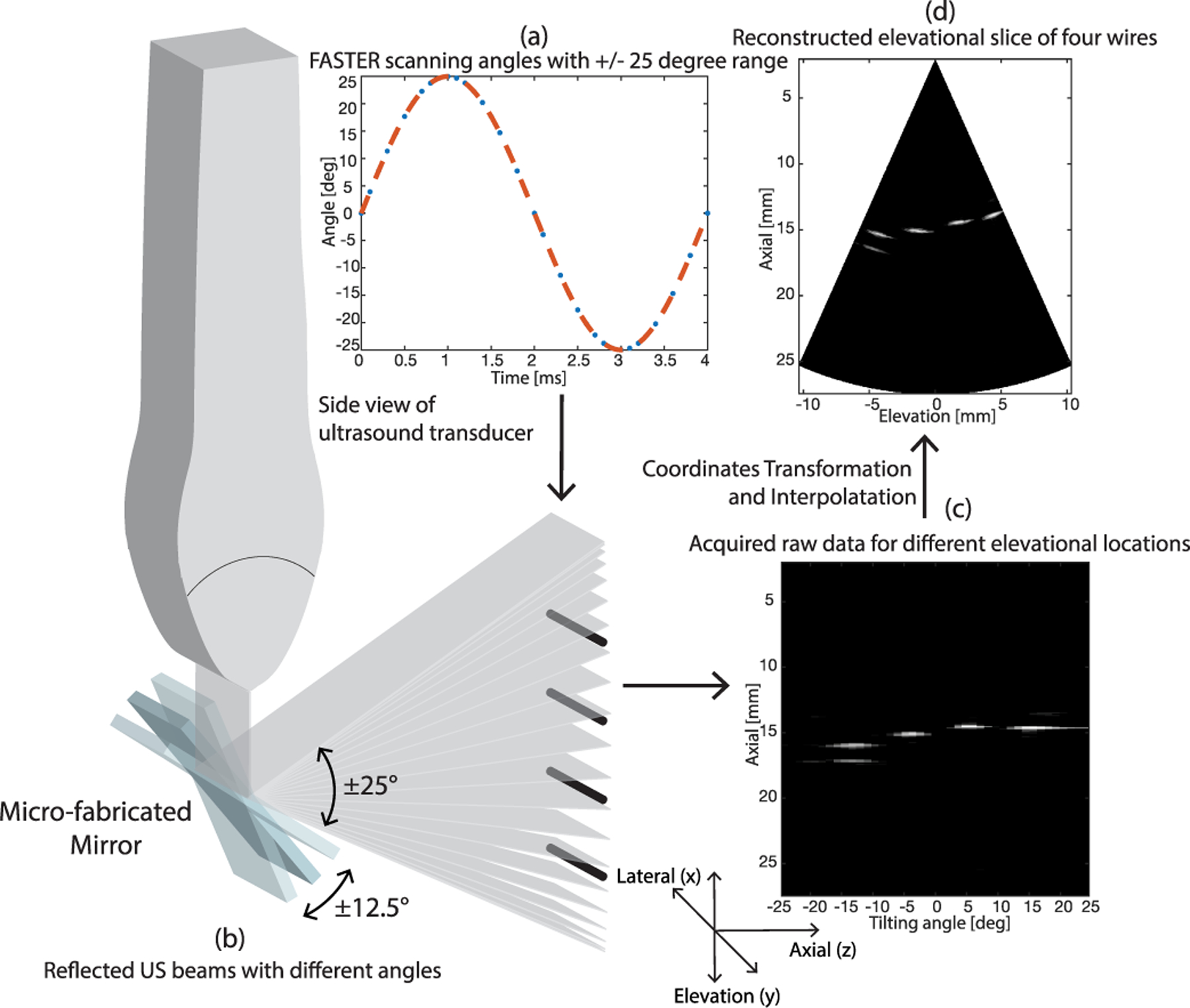
3-D image reconstruction scheme of FASTER 3-D imaging, a wire phantom is used for illustration. (a) Scanning angles with 25° half-side range. (b) Corresponding imaging planes reflected by the tilting microfabricated mirror. (c) Raw data (tilting angle versus axial dimension) sampled in the Polar coordinates. (d) Scan converted elevational–axial image (one imaging slice extracted from the 3-D volume).

The effective 3-D imaging average volume rate *F*_*v*_ is given by

(5) $$F_{v}=\{\begin{array}{c} 2F_{m},\;\;\text{condition }1\\ \frac{F_{m}}{m},\;\;\text{condition }2 \end{array}$$

where condition $1:=(\left\{\left({\text{PRF}}_{e}\right)/\left(F_{m}\right)\in A_{e}\right\}\cap \{\left({\text{PRF}}_{e}\right)/\left(F_{m}\cdot \pi \right)\phi \in \mathbb{Z}\})$, *A*_*e*_ denotes even number excluding imaging planes sampled at the largest scanning angles [e.g., ±25° in Fig. 1(a)]; condition 2 is the complement of the condition 1, and $m\in {\mathbb{Z}}^{+}$ is the smallest number when$\left(m{\text{PRF}}_{e}/F_{m}\right)\in {\mathbb{Z}}^{+}$. The factor of 2 in condition 1 comes from the observation that each spatial location is imaged twice during one tilting cycle of the mirror [see Fig. 1(a) and (b)].

Subsequently, the number of imaging planes (*N*_*p*_) sampled in one volume is

(6) $$N_{p}=\frac{{\text{PRF}}_{e}}{F_{v}}+1\left\{\left\{F_{v}=2F_{m}\right\}\cap {\text{C}}^{*}\right\}$$

where C*: When imaging planes at largest scanning angles were sampled, **1**{·} is the indicator function. The scan conversion for the *n*th imaging plane from the polar coordinates to the Cartesian coordinates can be expressed as

(7) $$y=d\;\text{sin}\;\alpha_{n}=d\;\text{sin}\;\left(2\theta_{n}\right)\;z=d\;\text{cos}\;\alpha_{n}=d\;\text{cos}\;\left(2\theta_{n}\right)$$

where *y* and *z* denote elevational and axial dimensions in the Cartesian coordinates, respectively, *d* is the imaging depth measured from the center of the microfabricated mirror plate (i.e., origin), and the lateral dimension (*x*) remains unchanged during scan conversion.

Equation (6) clearly shows that there is a tradeoff between the volume rate and the spatial sampling frequency in the elevational dimension: at a given PRF_*e*_, the higher the number of imaging planes (i.e., finer sampling along the elevational dimension), the lower the volume rate. For example, with an imaging depth of 3 cm, PRF can be as high 25 000 Hz with a single plane wave transmission, which leads to a volume rate of 500 Hz if *N*_*p*_ is 50. There also exists a tradeoff between the FOV and elevational line sampling from (4) and (7): at a given *N*_*p*_, the larger the scanning range (i.e., the larger FOV), the coarser the spatial resolution. Since the driving input signal to the mirror is sinusoidal [see (3)], the scanning angle of the mirror or the position of the imaging plane is not a linear function of time [see Fig. 1(a)]. Consequently, for accurate 3-D imaging, it is critical to synchronize the imaging sequence with the fast-tilting motion of the mirror and measure the instantaneous location of the mirror for robust 3-D reconstruction.

Based on the calibration measurements of the microfabricated mirror in air and in water (i.e., tilting angle range *A*, initial phase *ϕ*, and angle offset *γ*), 3-D image reconstruction can be conveniently performed by using standard scan conversion methods based on interpolation [8]. Fig. 1 illustrates the general workflow of FASTER. The transducer and the microfabricated mirror are synchronized by triggering the driving signal (generated by a function generator) of the microfabricated mirror with the ultrasound imaging system. The *n*th sampled imaging plane has a corresponding scanning angle *α*_*n*_ or tilting angle *θ*_*n*_ at *t*_*n*_ derived from (3) and (4), based on which the raw data sampled in the polar coordinate [see Fig. 1(c)] can be scan converted to the Cartesian coordinate [see Fig. 1(d)] using 3-D linear interpolation [29].

### B. Design and Fabrication of the Water-Immersible Microfabricated Scanning Mirror

**Fig. 2.**
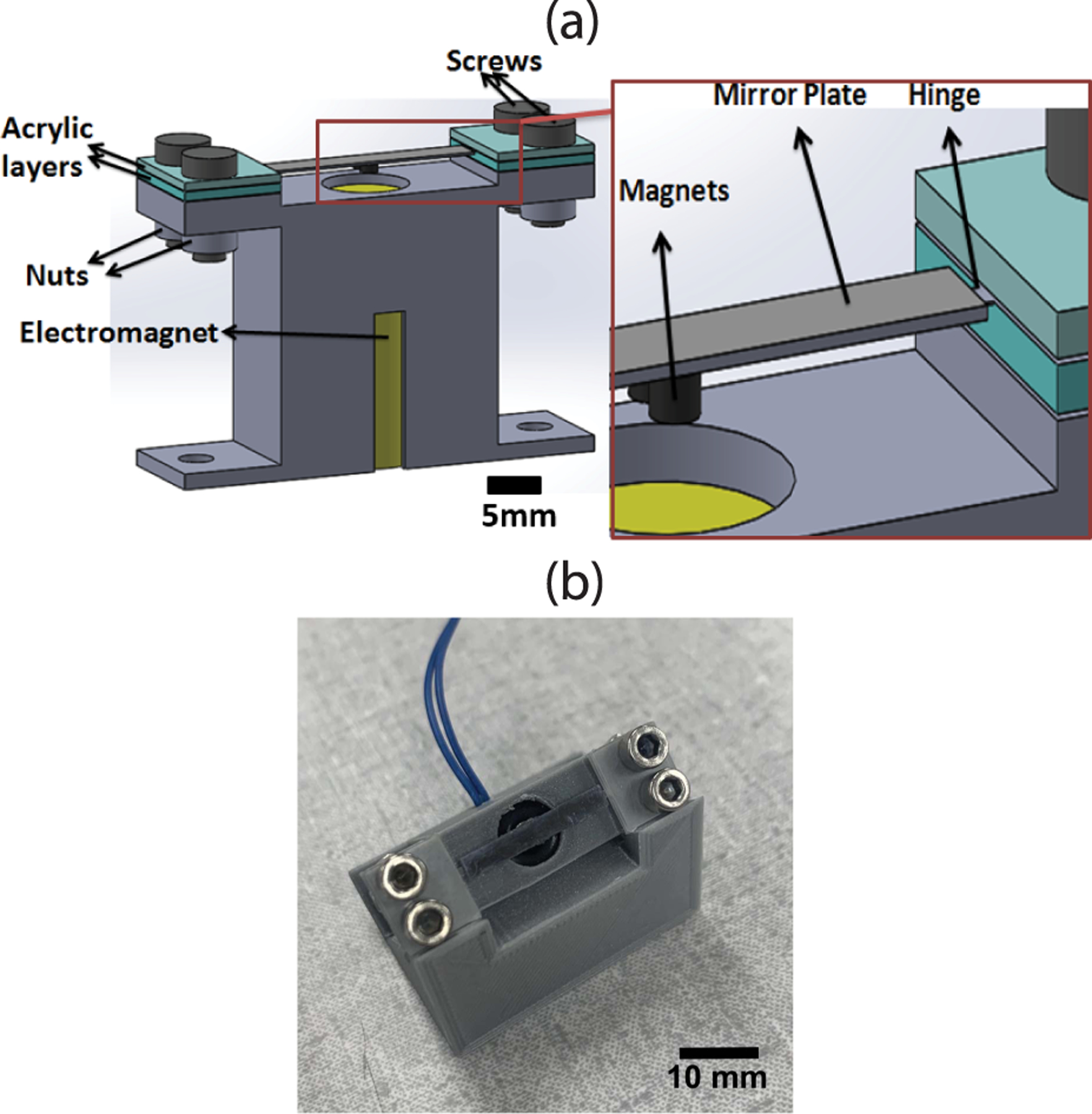
(a) Schematic design and (b) fabricated prototype of the water-immersible microfabricated scanning mirror.

The schematic design of the water-immersible microfabricated scanning mirror is shown in Fig. 2(a). The reflective mirror plate was made of a polished single-crystal wafer. The high acoustic impedance and the flatness of the silicon wafer provide high acoustic reflectivity and little distortion to the reflected ultrasound beam. The length and width of the mirror plate are made slightly larger than the focused ultrasound beam incident onto the mirror plate. The mirror plate is supported by two torsional polymer hinges made of biaxially oriented polyethylene terephthalate (BOPET). In contrast to the brittle silicon hinges oftentimes used in microfabricated scanning mirrors, the BOPET hinges have high tensile strength and chemical and dimensional stability. Therefore, they can well withstand the possible impact damage due to the shock or turbulence in water [30]. The two BOPET hinges are mechanically clamped onto a 3-D-printed plastic holder by four small screws. To enable the underwater scanning operation, electromagnetic actuation is selected as the driving mechanism for tilting the mirror plate around the torsional hinges. Compared with other actuation methods, electromagnetic actuation does not involve high voltage and, therefore, is more suitable for underwater operation [30]. Two microrare-earth magnet disks with opposite polarity are attached to two symmetric positions around the rotating axis at the center of the mirror plate. An electromagnet coil is assembled into the plastic holder, which is located right underneath the magnetic disks. When a direct current (dc) or alternating current (ac) is passing through the electromagnet coil, the magnetic field generated by the electromagnet coil creates a torque on the two magnet disks to tilt or vibrate the mirror plate around the torsional support structures. Fig. 2(b) shows the final fabricated water-immersible microfabricated scanning mirror, which has an overall dimension of 40.2 mm (*L*) × 11 mm (*W*) × 30.2 mm (*T*). Detailed design parameters are reported in Table I.

### C. Validation Study of Ultrasound Beam Reflections Based on the Microfabricated Mirror

**Fig. 3.**
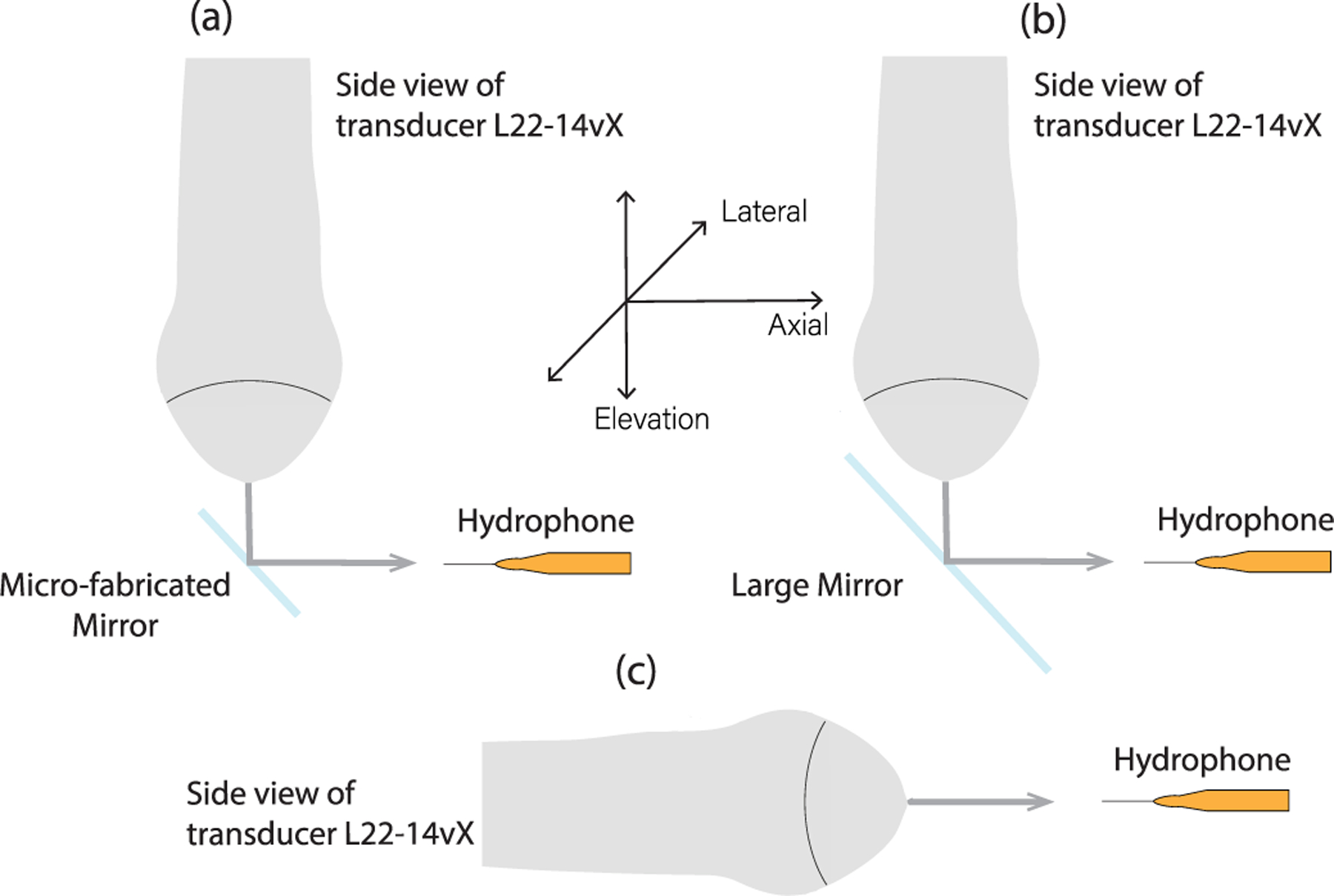
Experimental setup for the validation study of ultrasound beam reflection at 0° scanning angle. (a) Microfabricated mirror was used to redirect the ultrasound beam to the needle hydrophone. (b) Large mirror made from the same material as the microfabricated mirror plate was used for beam reflection. (c) Direct measurement from the transducer (without the mirror). The microfabricated mirror was not actively tilting during this experiment.

This part of the study was designed to validate the efficacy of beam reflection by the microfabricated mirror and examine if any beam distortion was introduced by the microfabricated mirror. A needle hydrophone (1603, Precision Acoustic Ltd., Dorset, U.K.) submerged in a water tank (filled with deionized water) was used to measure the acoustic pressure field in three different setups: 1) reflected by the microfabricated mirror; 2) reflected by a large mirror (84-mm diameter) that is made out of the same silicon wafer as in the microfabricated mirror; and 3) transmitted directly from the ultrasound transducer to the needle hydrophone without mirror reflection (see Fig. 3), and 0° and ±25° (only measured by the large mirror) scanning angles were studied. For all the experiments conducted in this study, an ultrafast plane wave imaging system Verasonics Vantage 256 (Verasonics Inc., Kirkland, WA) with a linear-array transducer L22-14vX (15.625 MHz, Verasonics, Inc., Kirkland, WA, USA) was used. The hydrophone was mounted on linear and rotary positioners (Daedal, Inc., Harrison City, PA, USA) to scan the 3-D acoustic field, with 0.1-, 2-, and 1-mm step size in elevational, axial, and lateral dimensions (2 mm in lateral dimension for the microfabricated mirror reflection), respectively. The Vantage system was synchronized with the needle hydrophone and the scanning stage.

By comparing the acoustic fields of the three experiment schemes, the beam profile can be systematically characterized to validate the feasibility of using a mirror reflector to conduct high volume rate 3-D ultrasound imaging.

### D. Optical Calibration Study of the Microfabricated Mirror

**Fig. 4.**
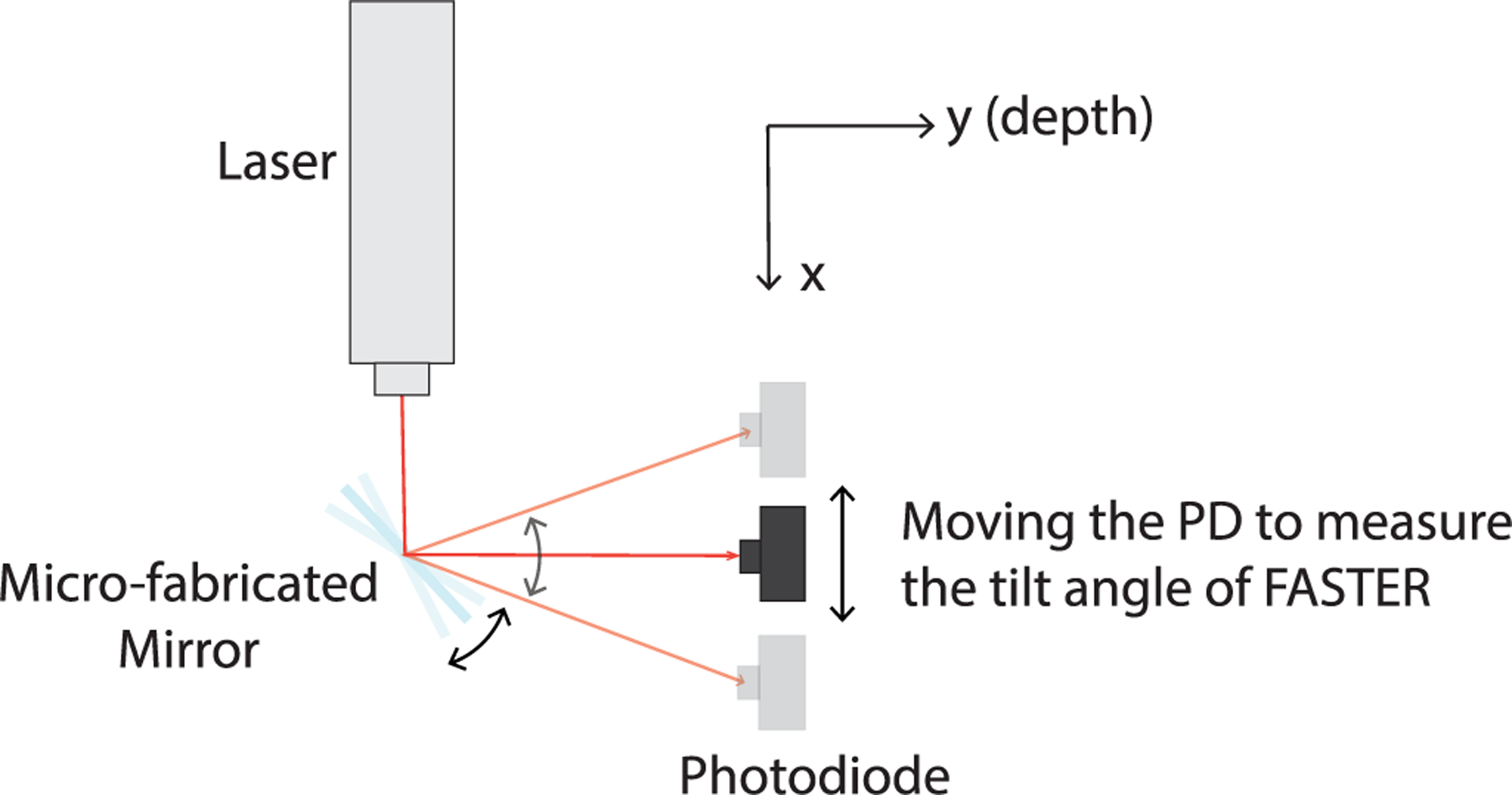
Experimental setup of the optical calibration study in air. A PD was used to measure the range of the laser beam reflected by the microfabricated mirror. The diameter of the laser beam is 2.5 mm.

Since the tilting frequency and tilting angle are essential for the accurate 3-D reconstruction of FASTER images, this part of the study was designed to use a laser and a photodiode (PD) to measure the tilting motion of the microfabricated mirror. The experiment setup is illustrated in Fig. 4. A 635-nm laser (CPS635R, ThorLabs, Inc., Newton, NJ, USA) was aligned with the center of the microfabricated mirror plate and received by a single point PD (DET10A, ThorLabs, Inc., Newton), which was mounted on a mechanical positioning system (KDC101, Thorlabs, Inc., Newton). The positioning system allowed accurate control of PD positioning along the *x*- and *y*-directions (see Fig. 4). All instruments were mounted on an optical table in this study. By moving along the *x*-direction, the range of the microfabricated mirror tilting angle can be measured. Adjusting the *y*-direction varies the distance between the microfabricated mirror and the PD. With this setup, the range of tilting angles at different driving voltages and frequencies can be calculated using simple geometry. To study the resonant frequency of the microfabricated mirror, the sinusoidal driving signal was fixed at 5 *V*_pp_ (peak-to-peak), and a frequency range of 100–350 Hz was tested with a 10-Hz step size. To establish the relationship between the tilting angle and driving voltage, the driving signal frequency was fixed at 250 Hz (the mechanical resonance frequency of the microfabricated mirror), and the voltage was varied from 10 mV_pp_ to 10 V_pp_ with a step size of 1 *V*_pp_.

### E. Acoustic Calibration Study of the Microfabricated Mirror

**Fig. 5.**
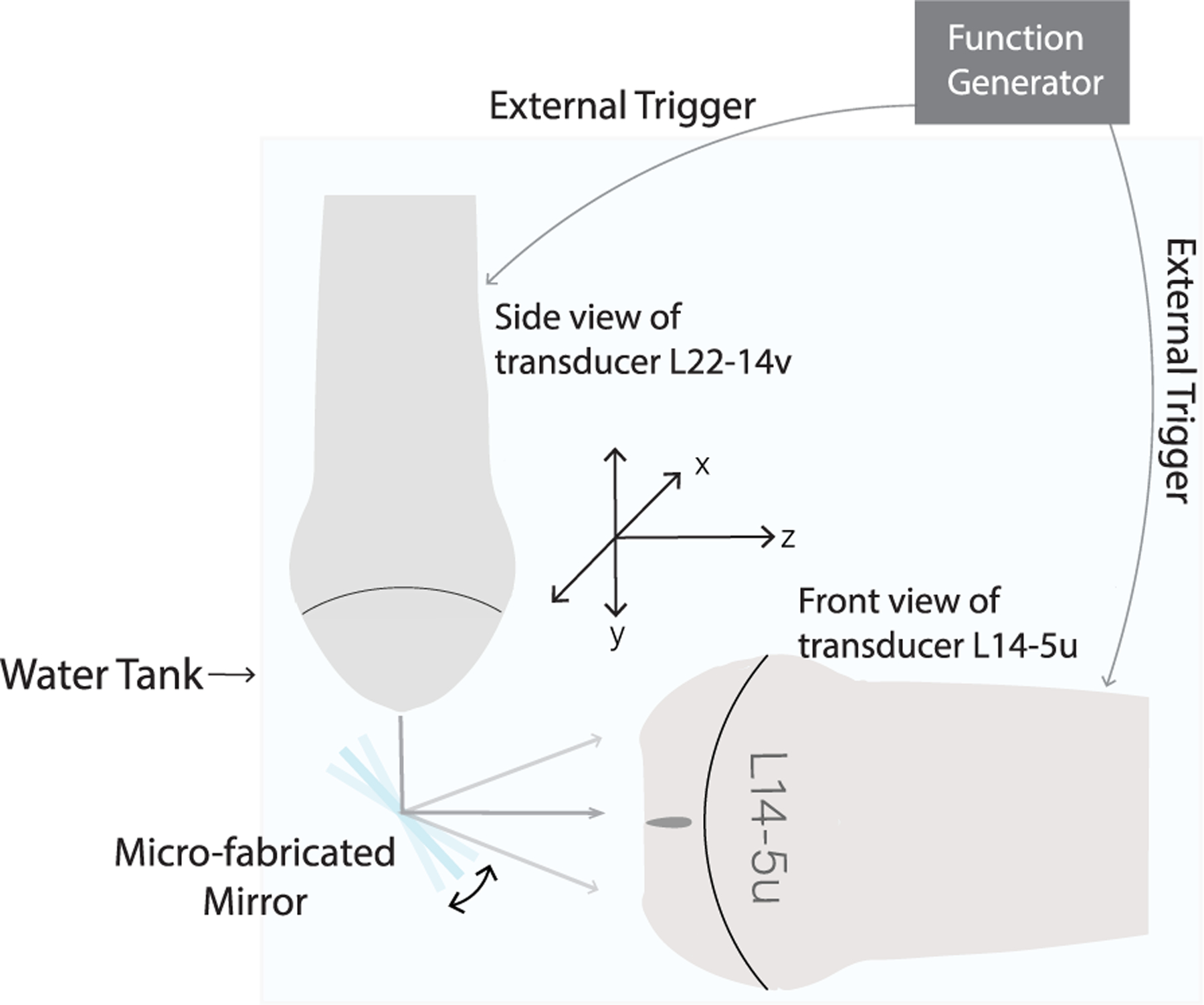
Experimental setup of the acoustic calibration study in water. The L22-14vX probe was used to transmit plane wave, the L14-5u probe was used to receive reflected ultrasound wave by the microfabricated mirror, and two probes were synchronized using a function generator.

This part of the study was designed to study the impact of water on the resonant frequency and tilting angle of the microfabricated mirror. Since most laser equipment and PDs are not designed for underwater applications, we resorted to using ultrasound for this part of the study. The experiment setup is illustrated in Fig. 5. The same L22-14vX transducer was used for transmission and an L14-5u (15.625 MHz, Ultrasonix Medical Corp., Burnaby, BC, Canada) was used for detection. Two separate Verasonics Vantage systems were used to drive the two transducers with synchronization via a function generator (33210A, Keysight Technologies Inc., Santa Rosa, CA, USA). The receiving transducer was controlled by the Daedal positioning system to move along the *x* and *z* dimensions to characterize the 4-D acoustic field (three spatial dimensions plus one temporal dimension) reflected by the tilting microfabricated mirror. Relations of the tilting angle with respect to the mirror driving frequency and voltage were characterized (the same parameters as in the optical calibration study). The measured range of mirror tilting angles [*A* in (3)], initial phase and tilting angle offset were used in 3-D image reconstruction.

### F. Phantom Study

**Fig. 6.**
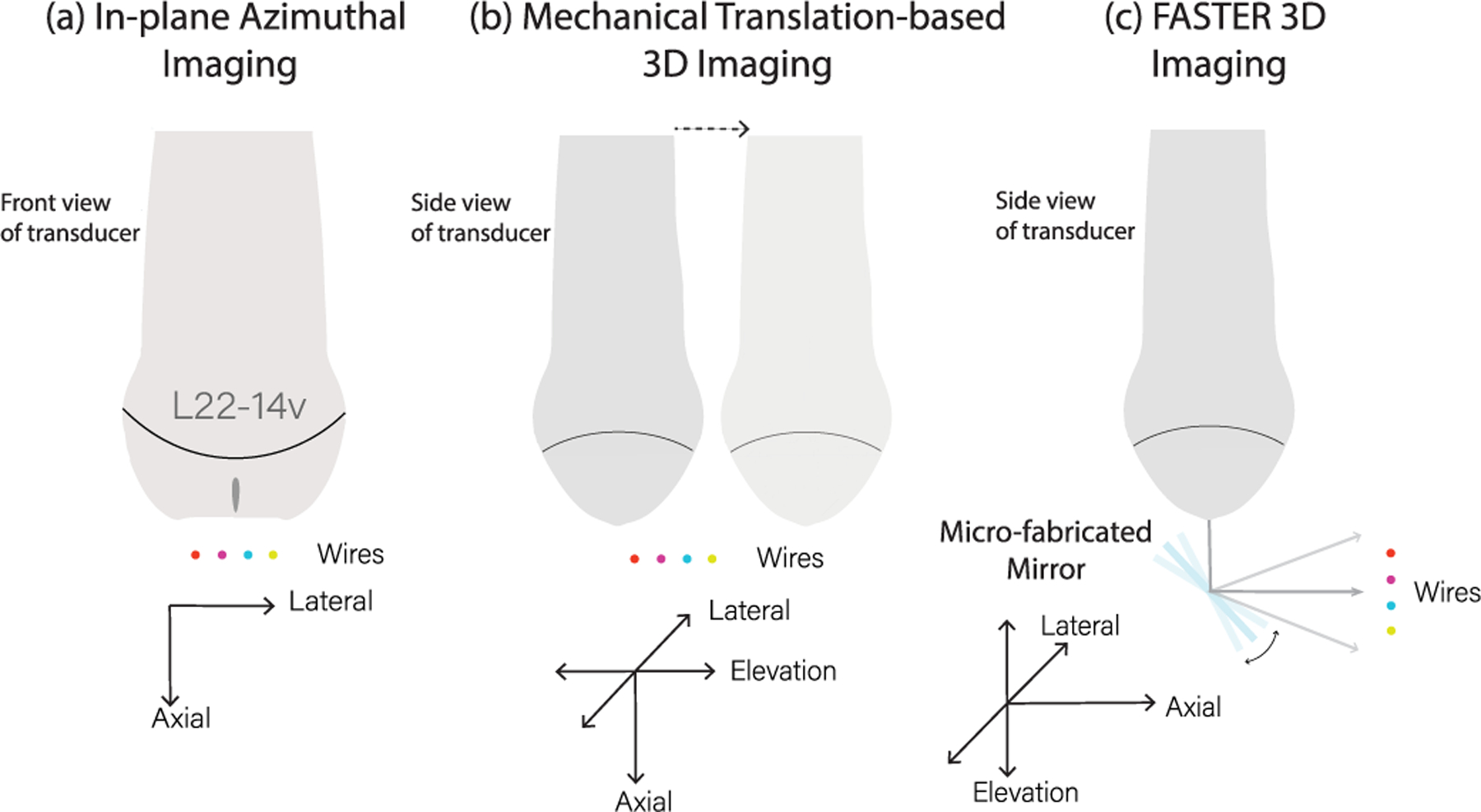
Experimental setup of three imaging methods. (a) In-plane azimuthal imaging. (b) Mechanical translation-based 3-D imaging. (c) FASTER 3-D imaging. In (c), the microfabricated mirror was driven by a function generator, which was synchronized with the imaging system.

To evaluate the performance of FASTER 3-D imaging, a wire phantom (tungsten 99.95%, 100-*μ*m diameter) was imaged in a water tank using the same L22-14vX transducer and the microfabricated mirror. For reference, the in-plane azimuthal imaging and mechanical translation-based 3-D imaging (i.e., similar to a wobbler) were performed to image the same set of wires using the same transducer. The azimuthal imaging was served as the reference of the true locations of the wires to test the spatial accuracy of FASTER 3-D imaging. The mechanical translation setup was used as a benchmark for 3-D imaging based on 1-D transducers. The experiment setup is shown in Fig. 6. For in-plane azimuthal imaging, four wires were imaged by the L22-14vX using a single plane wave transmission. For mechanical translation-based 3-D imaging, the mechanical translation was achieved by using the Daedal positioning system to translate the L22-14vX transducer with a 0.5-mm step size. The wire phantom consists of four wires with an approximately 3-mm distance between the wires. Various imaging depths from 10 to 25 mm with a 5-mm step size were tested. Detailed imaging configuration and parameters are summarized in Table II. Note that the mirror size in the elevation direction is larger than the beamwidth at the corresponding depth, which indicates a complete coverage of the incident ultrasound beam.

To evaluate the image contrast of FASTER 3-D imaging, a tissue-mimicking B-mode imaging phantom was made in-house with circular-shaped wall-less cavities at different locations to serve as anechoic targets. The phantom was imaged using a similar experimental setup as the wire phantom imaging. The in-plane azimuthal imaging and mechanical translation-based 3-D imaging were used for benchmarking with a single plane wave transmission. The contrast-to-noise ratio (CNR) was calculated as a quantitative metric to evaluate the image contrast of FASTER imaging. CNR was given by

(8) $$\text{CNR}=\frac{\left|{\bar{S}}_{t}-{\bar{S}}_{b}\right|}{\sqrt{\sigma_{t}^{2}+\sigma_{b}^{2}}}$$

where ${\bar{S}}_{t}$ and ${\bar{S}}_{b}$ are the mean values of the target and background, respectively, and $\sigma_{t}^{2}$ and $\sigma_{b}^{2}$ are the variances of the target and background, respectively.

## III. Results

### A. Validation Study of Ultrasound Beam Reflections Based on the Microfabricated Mirror

**Fig. 7.**
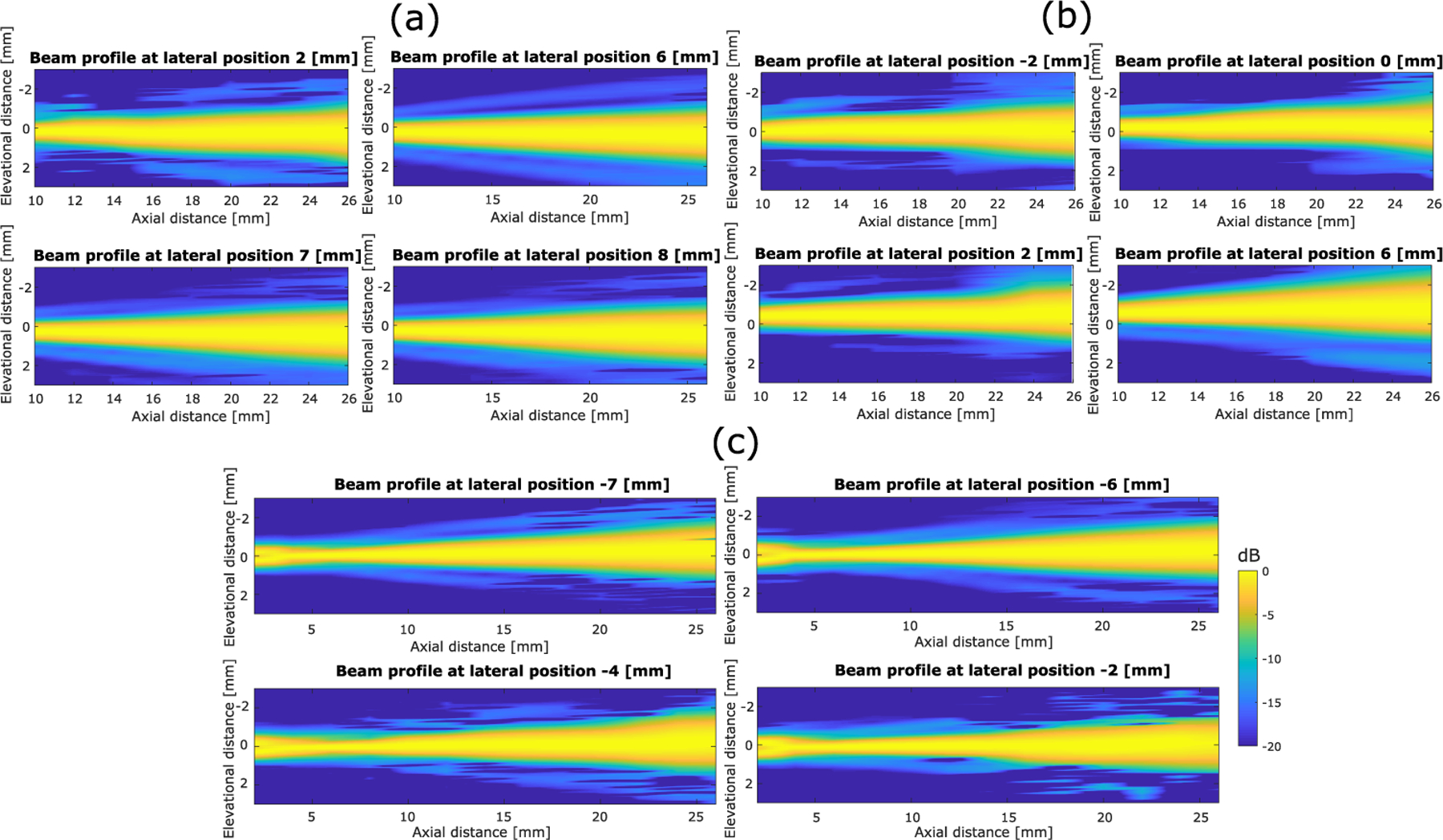
Representative beam profiles in elevational–axial orientation at different lateral locations for (a) large mirror reflection, (b) microfabricated mirror reflection, and (c) transducer only (no mirror reflection) when the scanning angle is 0°. All figures are in the same dynamic range of 20 dB. Low-pass filtering, windowing, linear interpolation along the axial and elevational direction (0.01-mm pixel size for both directions), and normalization along the elevational direction were implemented for better visualization.

**Fig. 8.**
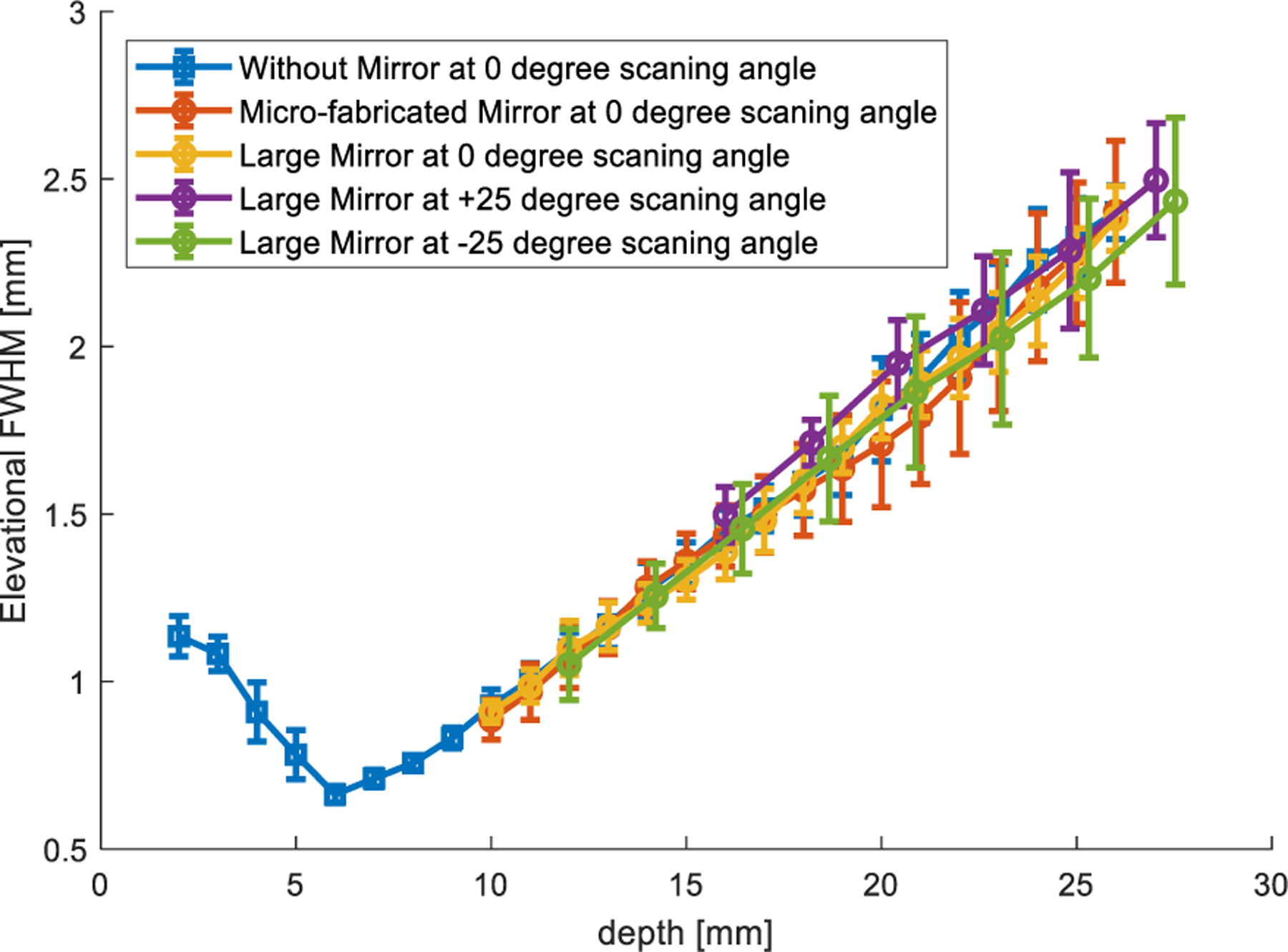
Elevational beamwidth measurements for three different scanning schemes (FASTER mirror reflection, large mirror reflection, and direct transmission without the mirror) at 0° and ±25° scanning angles.

**Fig. 9.**
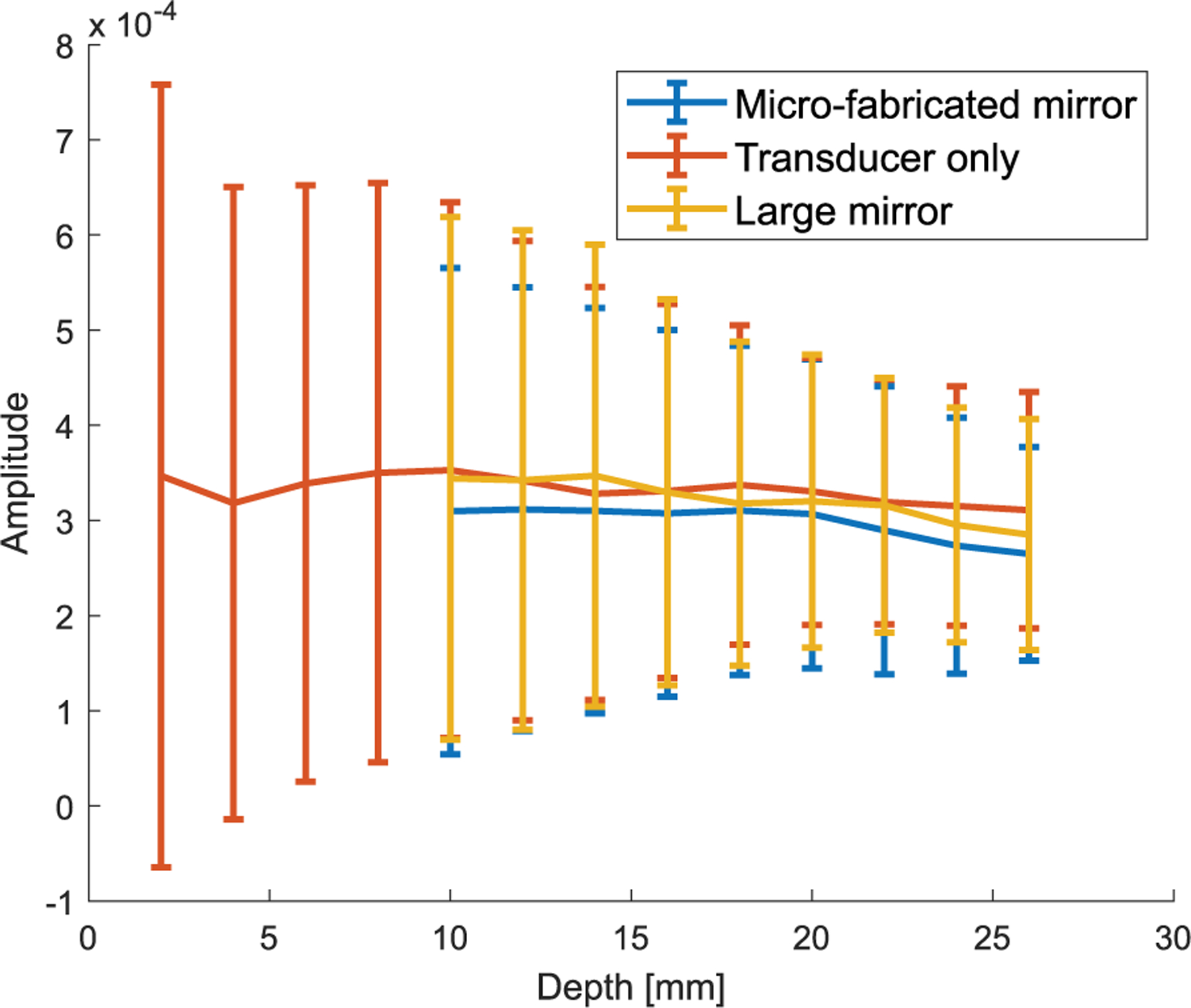
Amplitudes of hydrophone received signals at 0° scanning angle in three different schemes (microfabricated mirror, transducer only, and large mirror) and different depths; mean values at each depth were calculated using measurements across different elevational and lateral locations.

The acquired signal from the hydrophone at each location was preprocessed through averaging, low-pass filtering, and windowing. Then, the root-mean-square (rms) value was calculated to represent the acoustic intensity at each location. Full-width at half-maximum (FWHM) was used to calculate the elevational beamwidth. Fig. 7 shows the representative elevational beam profiles in the elevational–axial plane at different lateral locations when the scanning angle is 0° for the three scanning schemes: large mirror reflection, microfabricated mirror reflection, and direct transmission (i.e., without the mirror). For the setup without the mirror, a larger axial range was measured since the hydrophone could be positioned closer to the transducer surface. In Fig. 8, the mean and standard deviation of the elevational beam widths across different lateral locations and scanning angles were plotted against axial distance. Figs. 7 and 8 show that neither the microfabricated mirror nor the large mirror produced significant ultrasound beam distortions, and that the beamwidth measurements from using the mirrors were in good agreement with that from without using the mirror. Note that the elevational focus of the L22-14vX transducer was measured at approximately 6-mm depth, which is in good agreement with the specifications provided by the vendor [31]. Also, as shown in Fig. 9, reflections from the microfabricated mirror do not significantly alter the amplitude of the reflected signal, which is expected due to the high acoustic reflection coefficient of the silicon wafer used to fabricate the mirror.

### B. Optical Calibration Study of the Microfabricated Mirror

**Fig. 10.**
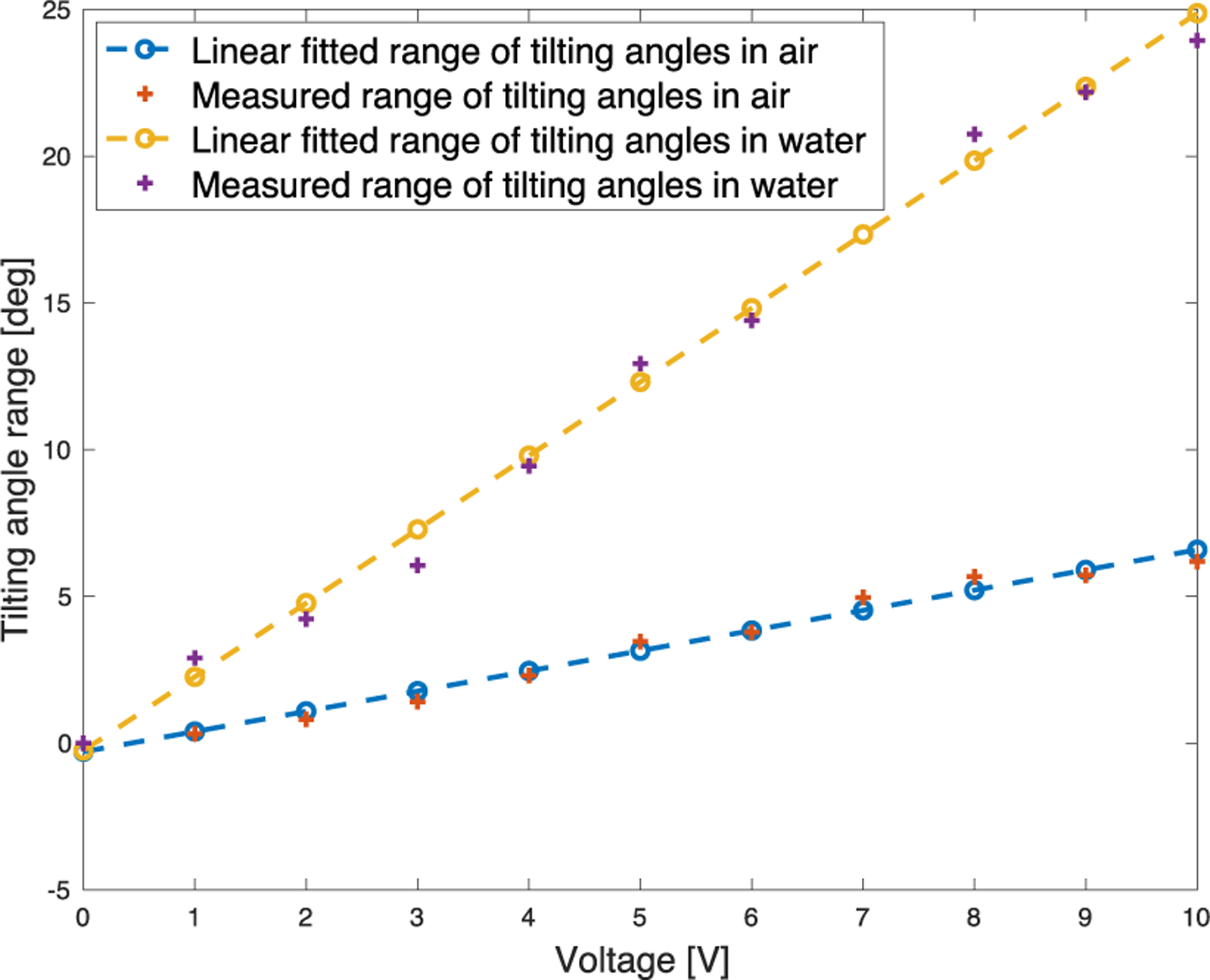
Relationship between the range of tilting angles of the microfabricated mirror and the driving voltage at 250-Hz tilting frequency measured in air and water.

**Fig. 11.**
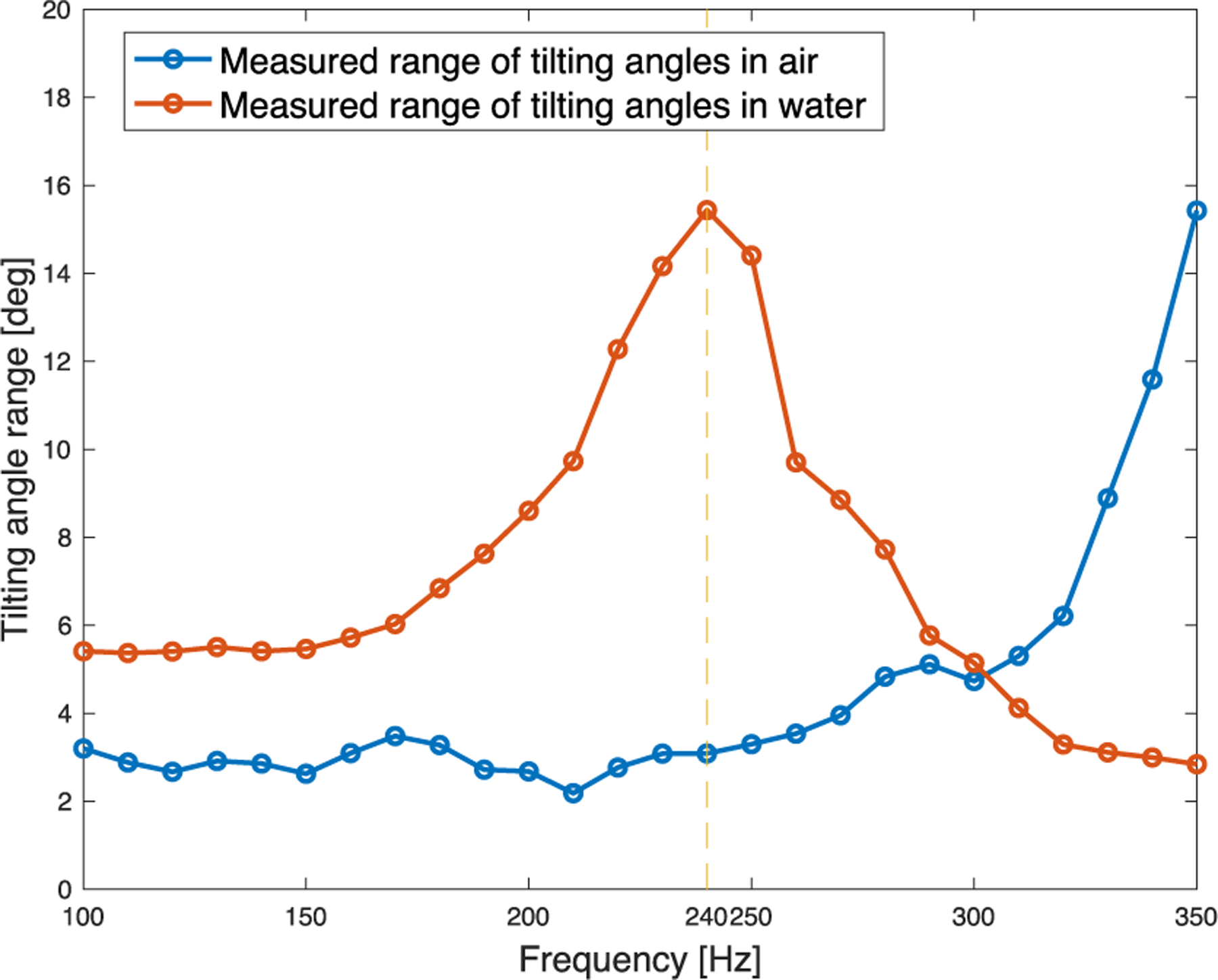
Measured tilting angle range with different tilting frequencies at 5 *V*_pp_ driving voltage in air and water.

Fig. 10 shows the total range of tilting angles (sum of two half-side tilting ranges) with respect to the driving voltage, with the frequency fixed at 250 Hz. As expected, we observed a linear relationship between the input signal amplitude and the microfabricated mirror tilting angle. This observation also holds in water (see Fig. 10). Fig. 11 shows that when operating in air, the resonant frequency of the microfabricated mirror is equal to or above 350 Hz. Measurements beyond 350 Hz were not conducted due to concerns of potential damage to the microfabricated mirror. Interestingly, as shown in Figs. 10 and 11, the microfabricated mirror has a significantly lower resonant frequency (~240 Hz) in water than in air, which is in favor of generating a large FOV in water. Based on our previous experiment, a downshift of resonant frequency on the scale of approximately 100 Hz is normal [32], [33]. However, these results indicate that it is critical to conduct the calibration experiment underwater to obtain the actual working characteristics for the microfabricated mirror.

### C. Acoustic Calibration Study of the Microfabricated Mirror

**Fig. 12.**
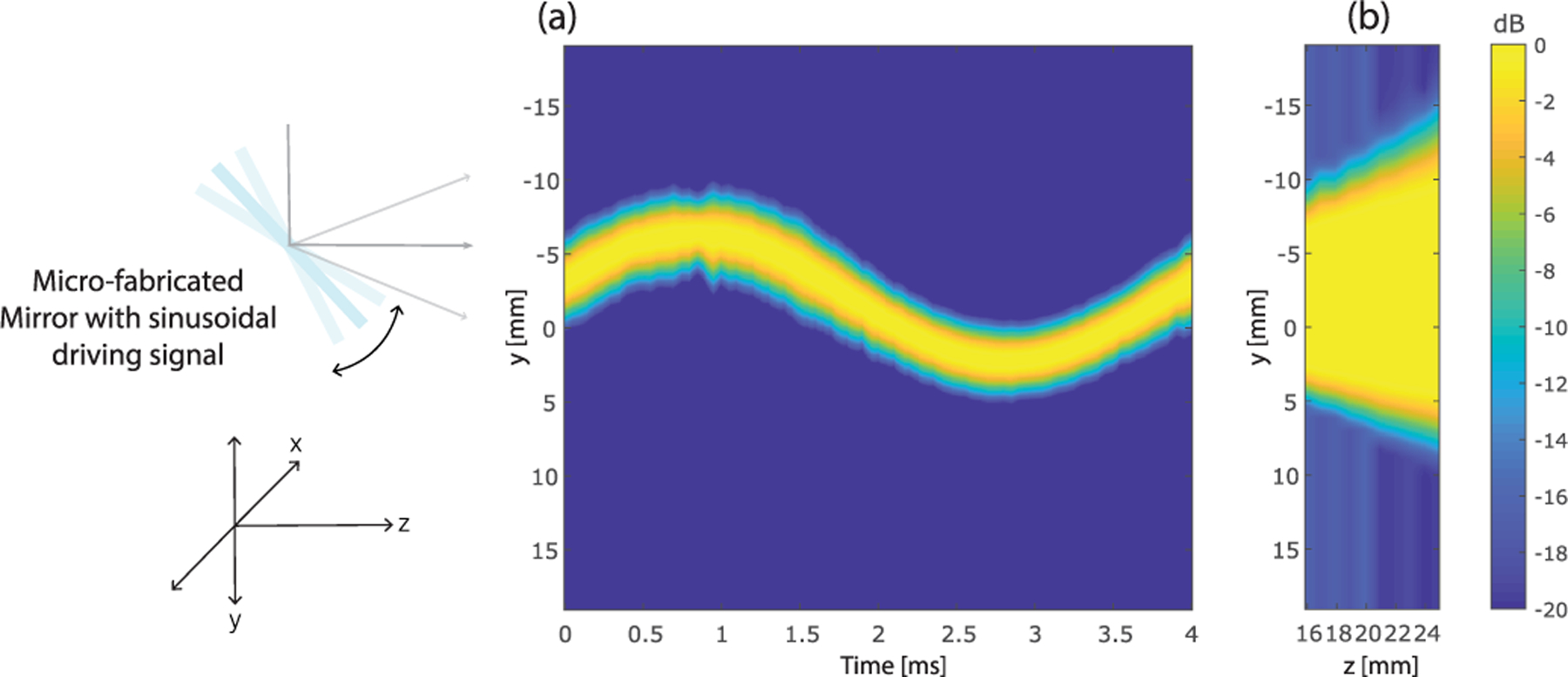
(a) Acoustically measured elevational beam intensity with respect to time. A clear sinusoidal-shaped elevational beam intensity profile was observed due to the scanning of the microfabricated mirror. The measurement was obtained from the center of the *x* dimension and at 16.9 mm *z*-position based on the coordinates shown in Fig. 5. (b) Peak beam intensity over time for each location of a 2-D *y*−*z* slice at the center of the *x* dimension. (a) and (b) are in the same dynamic range of 20 dB and with the same driving signal amplitude of 5 *V*_pp_.

Similar to the hydrophone signal processing in Section III-A, low-pass filtering and windowing were applied to preprocess the ultrasound channel data for each receive element of the L14-5u transducer, and then, the rms value of the radio frequency (RF) signal was used to represent the acoustic intensity at the corresponding position and time. Fig. 12(a) shows the beam intensity along the y dimension over time for a fixed *x*- and *z*- location. Clearly, the sinusoidal sweeping motion of the reflected beam is matched with the sinusoidal driving signal for the microfabricated mirror. The range of the tilting angles was calculated by measuring the peak intensity location of the elevational beam across 17 different *x* locations and ten different *z* locations. Fig. 12(b) shows the maximum acoustic intensity over time for each location (only a 2-D *y* − *z* slice is shown here from the central *x* location), from which we can identify the tilting range of the reflected acoustic beams.

### D. Phantom Study

**Fig. 13.**
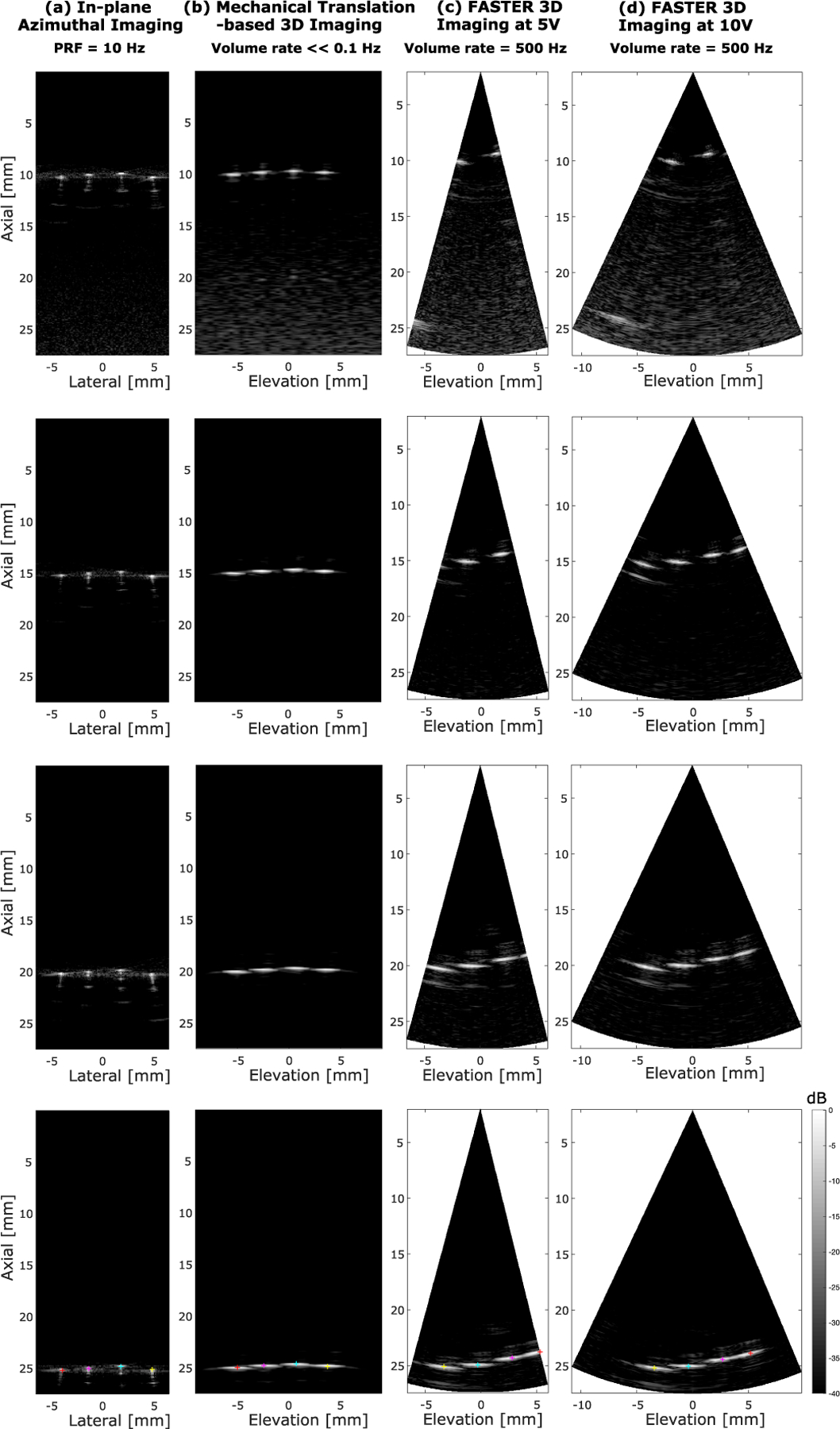
Azimuthal and elevational–axial images of the wire phantom placed at different axial depths with three different imaging methods. (a) In-plane azimuthal imaging. (b) Mechanical translation-based 3-D imaging. (c) FASTER imaging with a driving voltage of 5 *V*_pp_. (d) FASTER imaging with a driving voltage of 10 *V*_pp_. Different rows show images of the wires at different axial locations. The wires are marked with different colors at the bottom row for testing the accuracy of imaging the wire positions. All images are in the same dynamic range of 40 dB.

**Fig. 14.**
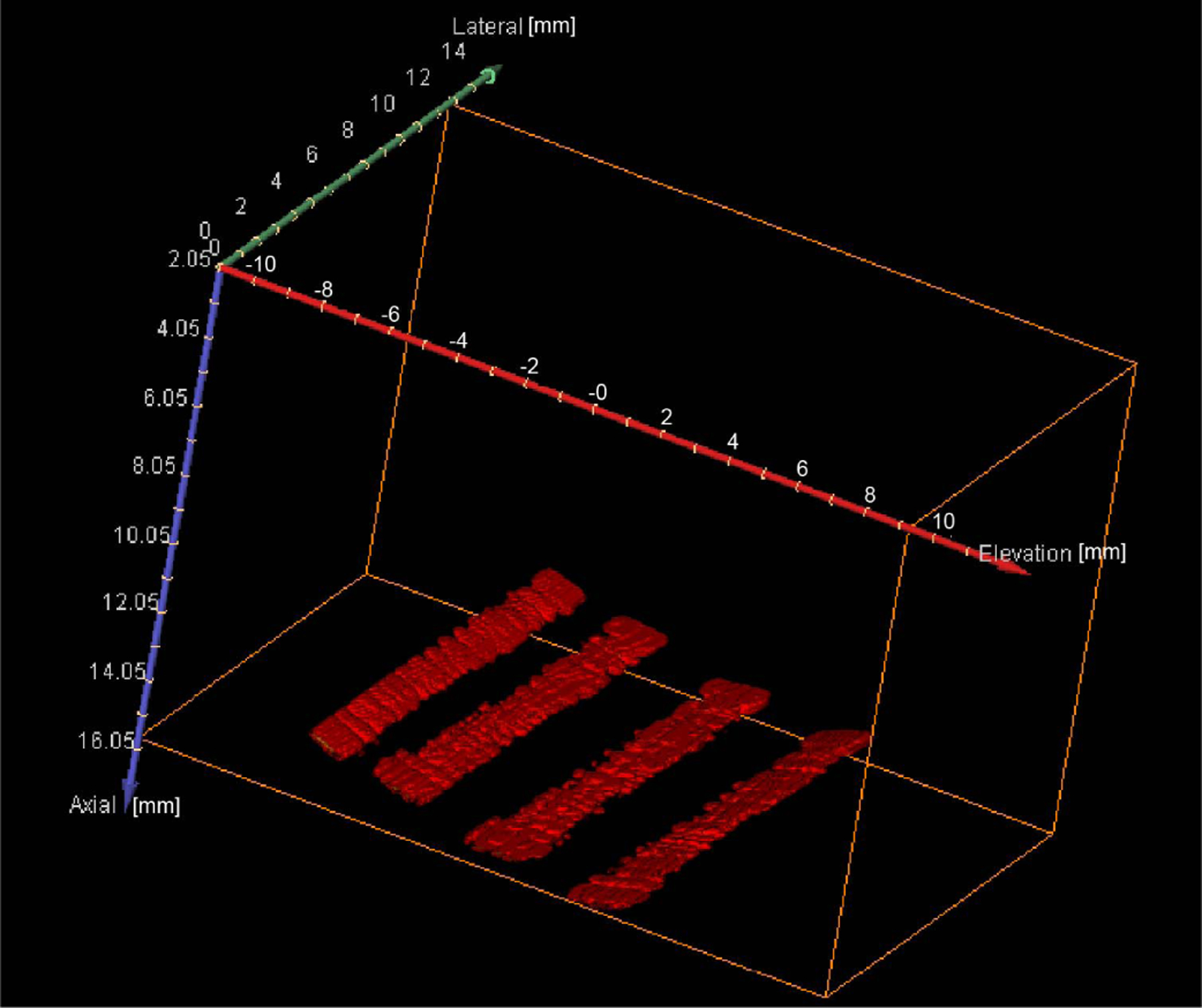
Reconstructed volumetric image of the wire phantom from FASTER 3-D imaging. The four wires were positioned at 15-mm axial depth and the driving voltage of the microfabricated mirror was 10 *V*_pp_. The dynamic range is 20 dB.

Fig. 13 shows the azimuthal and elevational–axial images of the cross section of the four wires at different axial depths by the three different imaging schemes (in-plane azimuthal imaging, mechanical translation-based 3-D imaging, and FASTER 3-D imaging). The positions of the four wires were extracted from the azimuthal images [column under (a)] and marked by markers with different colors (only shown in the bottom row for succinctness). The actual locations of the four wires in the two 3-D imaging schemes were derived through spatial translations and rotations of the extracted positions in Fig. 13(a) because relative positions of the wires with respect to the transducer were changed during the experiment. From Fig. 13, it is clear that the lateral resolution deteriorates with depth due to increased beamwidth in the elevational direction. However, the four wires remain clearly separable at 25-mm depth using both mechanical translation and FASTER 3-D imaging. Meanwhile, FASTER was able to construct 3-D images with accurate wire locations (results not shown for depths from 10 to 20 mm). The final reconstructed 3-D images of the wires by FASTER are shown in Fig. 14. Note that the wire phantom was made in-house; therefore, the four wires were not strictly aligned at the same depth.

**Fig. 15.**
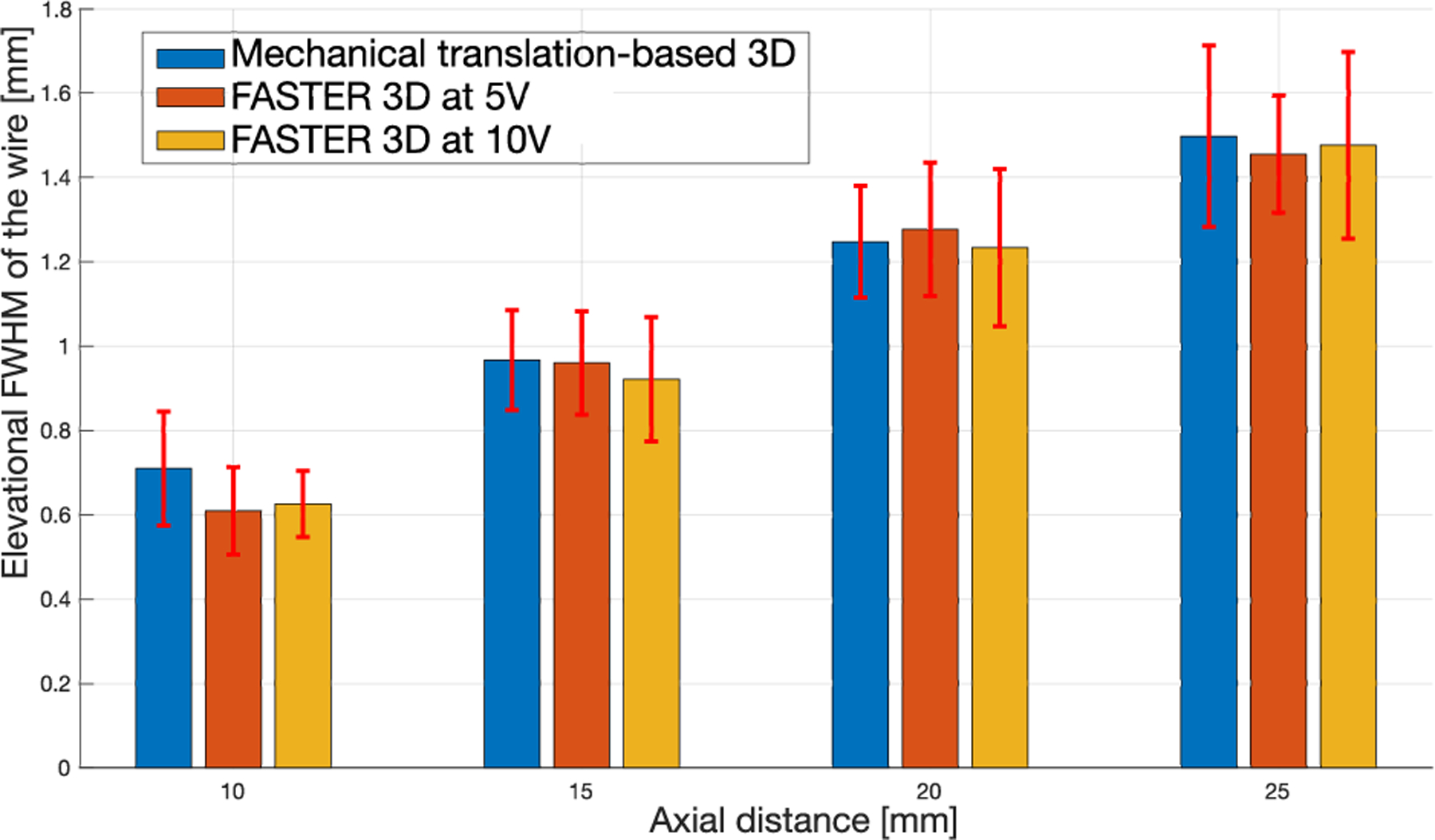
Summary plots of the measured elevational resolution of the wire of FASTER 3-D imaging and mechanical translation-based 3-D imaging.

Fig. 15 summarizes the elevational resolutions of various setups at different imaging depths. The results show that FASTER provides comparable elevational imaging resolution with the mechanical translation-based 3-D imaging technique.

**Fig. 16.**
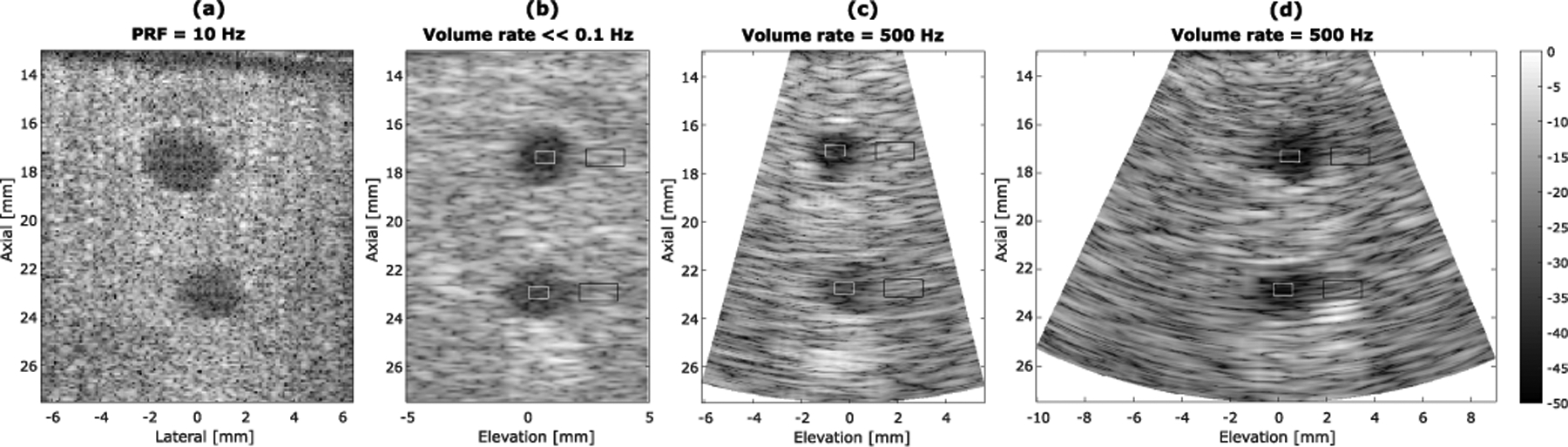
Azimuthal and elevational–axial images of the tissue-mimicking phantom with anechoic targets placed at around 17- and 23-mm depths using three different imaging methods. (a) In-plane azimuthal imaging. (b) Mechanical translation-based 3-D imaging. (c) FASTER imaging with a driving voltage of 5 *V*_pp_. (d) FASTER imaging with a driving voltage of 10 *V*_pp_. Regions of interest for the target (white frames) and regions of interest for the background (black frames) are labeled. All images are with the same dynamic range of 50 dB.

**Fig. 17.**
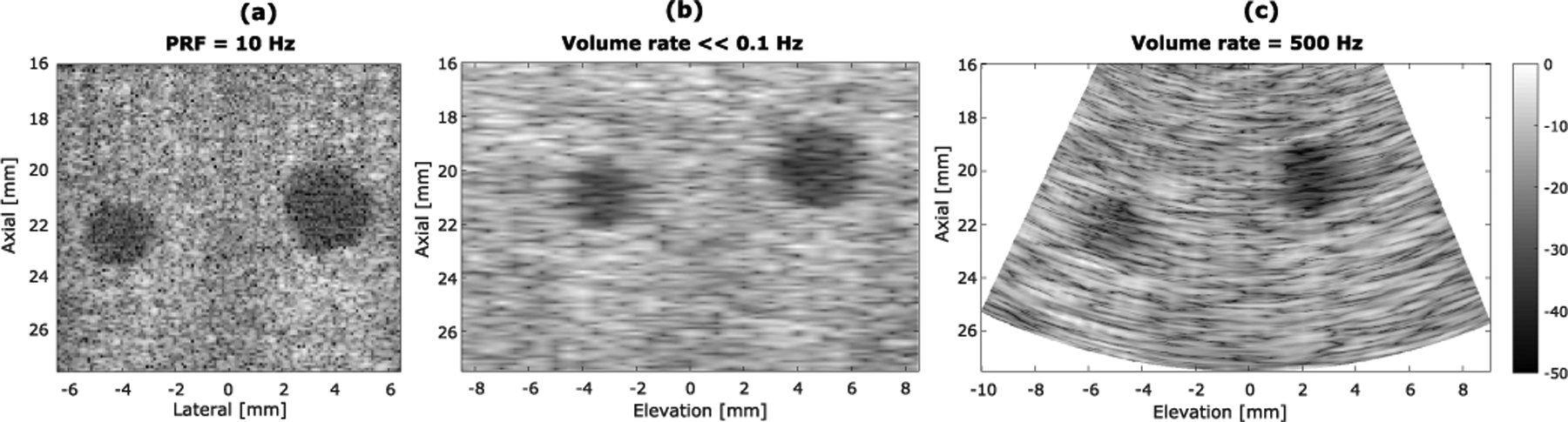
Azimuthal and elevational–axial images of the tissue-mimicking phantom with anechoic targets placed at different lateral locations using three different imaging methods. (a) In-plane azimuthal imaging. (b) Mechanical translation-based 3-D imaging. (c) FASTER imaging with a driving voltage of 10 *V*_pp_. All images are in the same dynamic range of 50 dB.

Fig. 16 shows the azimuthal and elevational–axial images of the cross section of the two targets at different axial depths by the three different imaging schemes (in-plane azimuthal imaging, mechanical translation-based 3-D imaging, and FASTER 3-D imaging). Similarly, Fig. 17 shows the images of two targets at different lateral locations.

Region of interests (ROIs) for CNR measurements were indicated in Fig. 16. CNR [see (8)] with measurements summarized in Table III. The contrast (i.e.,$\left|{\bar{S}}_{t}-{\bar{S}}_{b}\right|$) is similar between conventional mechanical translation-based 3-D imaging and FASTER 3-D imaging. The standard deviation of the target region and background region for mechanical translation-based 3-D imaging is smaller than those of FASTER 3-D imaging. This is because a smaller translation step size was used for mechanical translation-based 3-D imaging (i.e., 0.2 mm), which contributes to a reduced variance of the image.

## IV. Discussion

Currently, 3-D ultrasound imaging is challenged by various technical limitations of existing techniques, such as limited volume rate for 1-D transducer-based 3-D scanning, high complexity and costs associated with 2-D ultrasound transducers, and suboptimal spatial resolution and imaging quality for emerging techniques, such as RCA transducers and sparse arrays. These technical challenges remain as major hurdles for effectively translating 3-D ultrasound imaging into clinical practice. In this article, we introduced a new method of conducting high volume rate 3-D imaging—FASTER—which is based on a fast-tilting, water-immersible microfabricated mirror and conventional 1-D ultrasound transducers. We demonstrated robust imaging performance of FASTER with high imaging volume rate and comparable spatial resolution with conventional mechanical translation-based 3-D imaging methods. The validation study shows that mirror reflection does not distort the ultrasound beam, which verifies the feasibility of using a reflector to conduct accurate 3-D imaging. We showed that the tilting angle and tilting range of the microfabricated mirror could be characterized in air and in water, which can be used for robust 3-D reconstruction of FASTER images. The wire phantom experiment shows that FASTER 3-D can accurately reconstruct the imaging targets with comparable spatial resolution and substantially higher imaging volume rate over the conventional mechanical translation-based 3-D imaging.

One main advantage of FASTER 3-D compared with mainstream 3-D ultrasound techniques is the high imaging volume rate realized at relatively low fabrication and computational costs. The microfabricated mirror enjoys a small form factor and can be conveniently combined with conventional 1-D ultrasound transducers to perform 3-D imaging on a 2-D ultrasound imaging system that, otherwise, does not support 3-D imaging. Another advantage of FASTER 3-D imaging is the relatively low beamforming computational cost compared with fully populated 2-D transducers since no elevational beamforming is needed. The downside, however, is that FASTER 3-D does not support transmit or receive focusing in the elevational direction, such as a 2-D transducer, hence lower spatial resolution and signal-to-noise ratio (SNR). Nevertheless, the in-plane azimuthal imaging quality of FASTER is essentially identical to that of conventional 2-D plane wave ultrasound imaging, which can be significantly better than that from 2-D ultrasound transducers. Although FASTER 3-D involves a considerable amount of interpolation computations for the scan conversion process, the cost can be effectively alleviated by parallel computing based on a graphics processing unit (GPU).

**Fig. 18.**
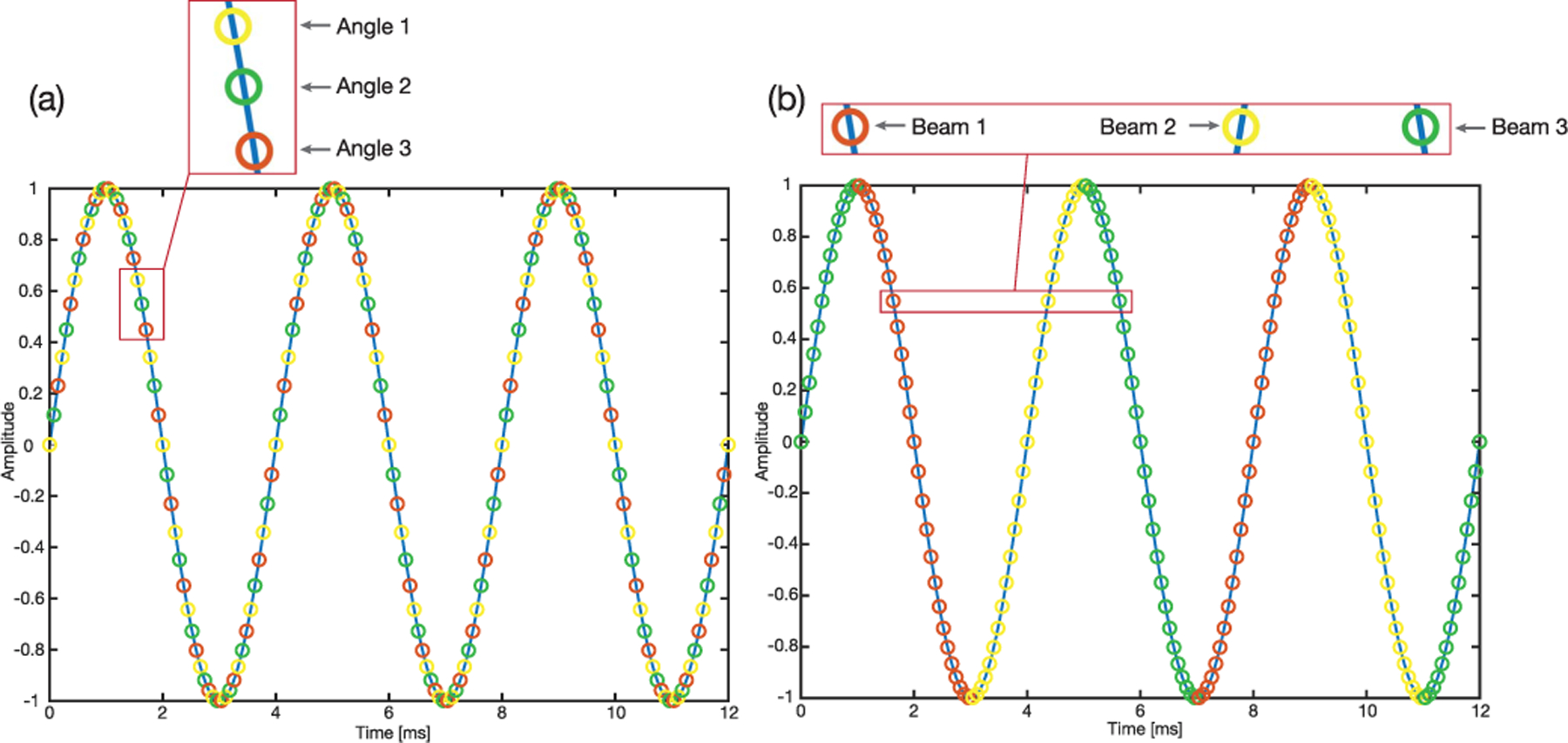
(a) Compounding plane wave imaging scheme, three different angles are transmitted continuously. (b) Focused-beam-based line-byline scanning scheme, three different beams are transmitted in different sweeps. The Blue solid line represents a 250-Hz sinusoidal driving signal, and circles represent sampled tilting angles (PRF is 13 500 Hz) or sampled elevational positions, and three different colors of circles represent three different compounding angles or focused beams.

FASTER enjoys high azimuthal image quality in a sense that FASTER can achieve the same high-quality azimuthal imaging as traditional 1-D transducers. For example, one can use compounding plane wave imaging or focused-beam-based line-by-line scanning to boost the imaging quality. However, the tradeoff is a reduced imaging volume or reduced number of elevational sampled positions because of the increased number of pulse–echo cycles needed for line-by-line scanning and compounding (as demonstrated in Fig. 18). There are two different methods: 1) for compounding, different angles are transmitted continuously, and volume rate stays the same with plane wave imaging, while the number of sampled locations is reduced by a factor of the number of angles; note that the image may be blurred due to several angles acquired from different locations are combined and 2) for line-by-line scanning, different beams are transmitted in different sweeps, volume rate is reduced by the factor of the number of beams, and the number of sampled locations maintains the same. Only three angles or beams were used in Fig. 18 for the purpose of illustration, and more angles or beams can be used in practice to achieve high azimuthal image quality. Also, the elevational imaging resolution can be improved through the synthetic aperture method [34]. Assume that *F*_*v*_ [see (5)] is the volume rate when plane wave imaging is applied and *N*_*p*_ [see (6)] is the corresponding imaging planes sampled; then, for the focused-beam-based line-by-line scanning, the volume rate is reduced to

(9) $$F_{v,l}=\frac{F_{v}}{n_{l}}$$

where *n*_*l*_ is the number of scanning lines or focused beams. The imaging plane sampled of the line-by-line scanning stays the same

(10) $$N_{p,l}=N_{p}.$$

Another limitation of the study is that a sinusoidal driving input signal was used, which does not provide a linear function of time for uniform sampling. A triangular driving input signal would have provided a linear sampling of the scanning range (i.e., locations), which is superior to the sinusoidal signal. However, since the microfabricated mirror is a narrow-band device, even with a triangular input signal, the output remains a sinusoidal wave.

**Fig. 19.**
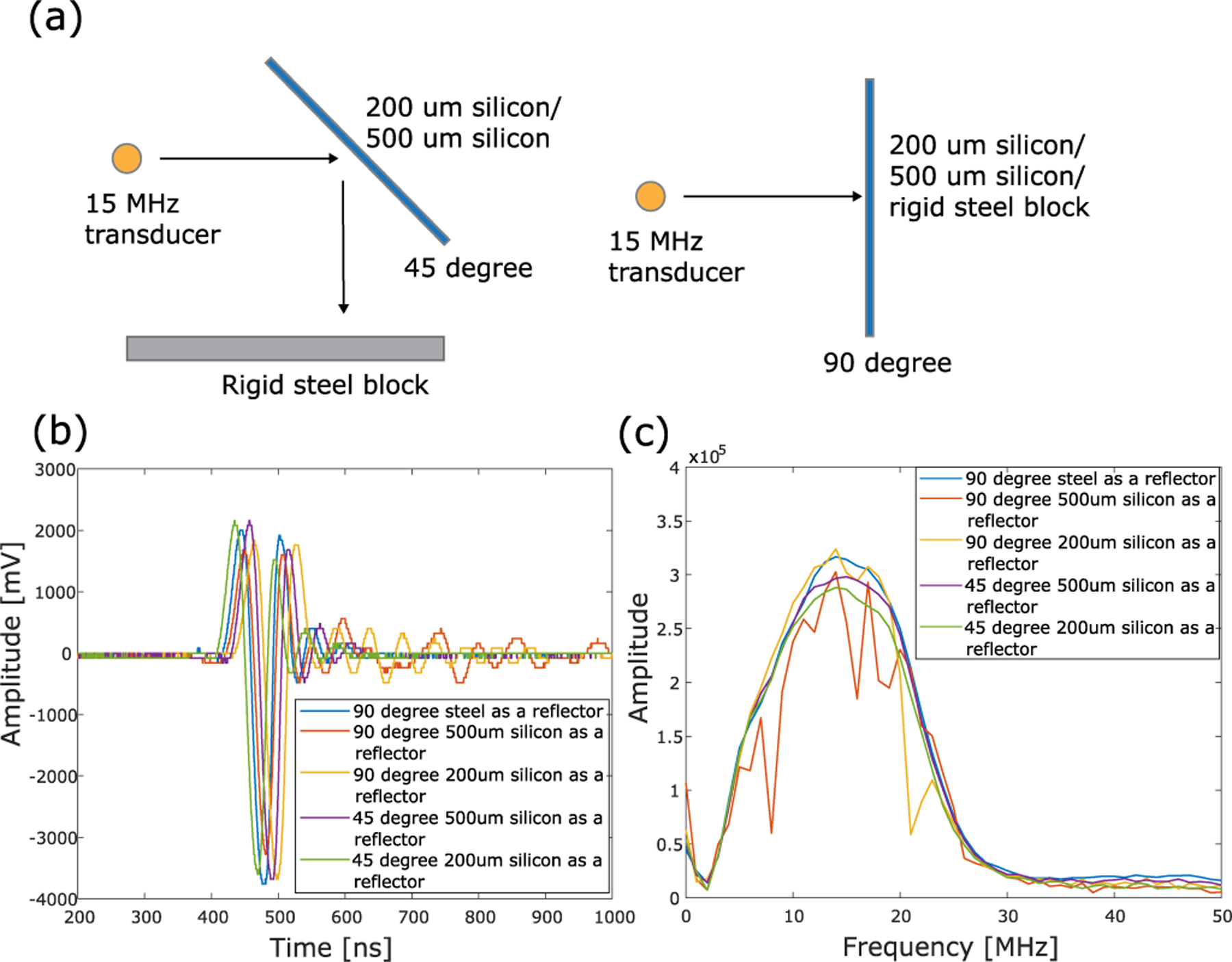
(a) Experimental setup to measure the reflection spectrum of the silicon wafer with different thicknesses (200 *μ*m and 500 *μ*m) under different incident angles: 45° incident angle (left) and 90° incident angle (right). A rigid steel block was used to reflect the transmitted signal to the same transducer as the receiver. (b) Reflected signals in the time domain using different incident angles (45° and 90°) and different reflectors (200-*μ*m silicon, 500-*μ*m silicon, and a rigid steel block). (c) Spectra from different incident angles (45° and 90°) and different reflectors (200-*μ*m silicon, 500-*μ*m silicon, and a rigid steel block).

To determine the influence of wafer thickness-mode resonant frequencies on the TX/RX bandwidth, an experiment was conducted to measure the reflection spectrum using 200- and 500-um silicon wafers, a rigid steel block as reflectors, and a 15-MHz focused transducer [see Fig. 19(a)]. Results of spectrums using different incident angles (45° and 90°) and different reflectors (200-*μ*m silicon, 500-*μ*m silicon, and rigid steel block) are shown in Fig. 19(c), and the corresponding reflected signals in the time domain were shown in Fig. 19(b). From that, we can derive that the bandwidths using 200- and 500-*μ*m silicon maintain the same compared with the reflection spectrum using the rigid steel block. Also, the amplitudes of the signals maintain at approximately the same level, which is matched with the observation in Fig. 9 and indicates that reflection does not significantly change the amplitude of the signal.

There are several tradeoffs that need to be balanced when using FASTER 3-D. First, to achieve a high imaging volume rate, one needs to increase the tilting frequency of the mirror [see (5)]. However, at a given maximum PRF, increasing the mirror tilting frequency would lead to a decrease in elevational sampling positions (i.e., increased imaging plane spacing and coarser pixel resolution). If the imaging plane spacing needs to be maintained, a smaller tilting angle range (i.e., smaller FOV) needs to be implemented to accommodate for the increase in imaging volume rate. Second, to achieve a large imaging volume, one needs to increase the tilting angle range of the mirror [i.e., *A* in (3)]. If the fixed imaging plane spacing is desired in the elevational direction, then the number of imaging planes [i.e., *Np* in (6)] needs to increase, which leads to a decreased imaging volume rate if PRF cannot be elevated correspondingly. Third, to improve the in-plane imaging quality (e.g., imaging resolution and SNR), techniques such as spatial compounding and line-by-line focused or wide beam scanning are needed, which leads to a decreased effective PRF and, consequently, imaging volume rate. Consequently, the imaging volume and/or the elevational sampling positions will need to be decreased to compensate for the loss in the imaging volume rate. This may be necessary for applications such as blood flow imaging and shear wave elastography where a high volume rate is needed for motion tracking. For B-mode imaging, however, a volume rate of 10–20 Hz may be adequate for real-time scanning, which can be easily fulfilled by the high volume rate offered by FASTER even if a large number of compounding angles and focused scan lines are being implemented. Meanwhile, to improve elevational imaging resolution, an acoustic lens can be added in between the ultrasound transducer and the microfabricated mirror or between the microfabricated mirror and the tissue to focus the beam in the elevational dimension.

**Fig. 20.**
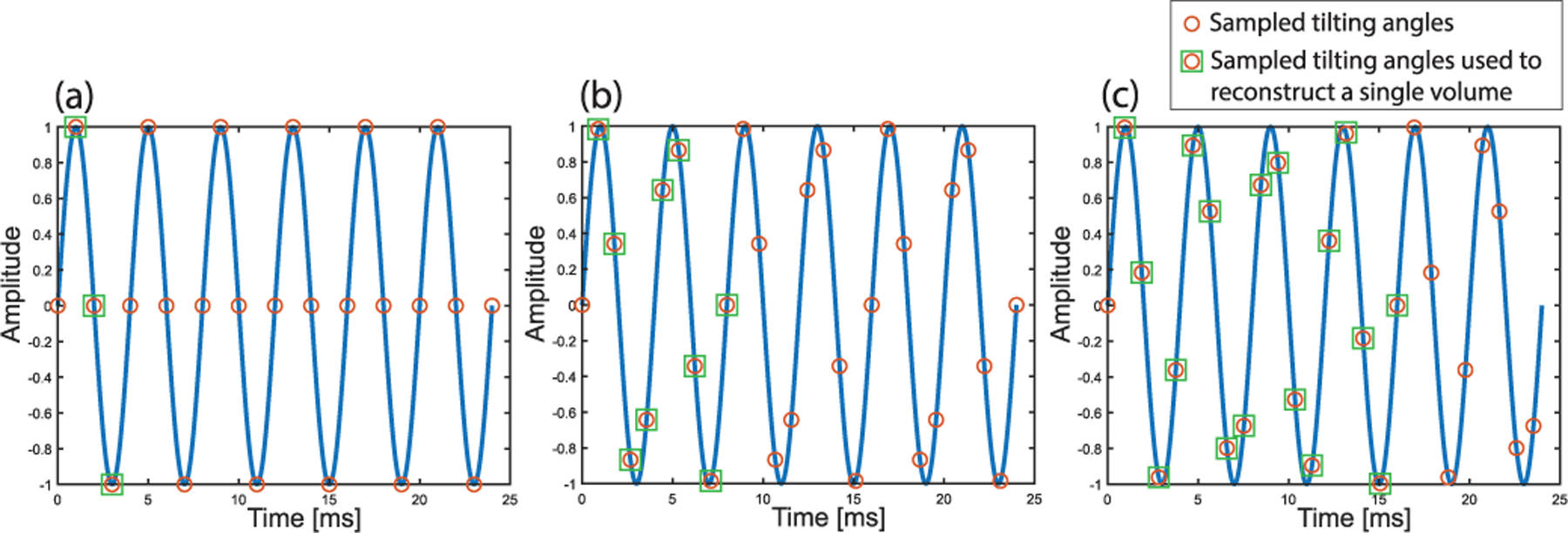
Sinusoidal driving signal (blue line) at 250 Hz in six cycles and sampled tilting angles (orange circle) with different pulse repetition frequencies (PRF) and corresponding volume rates. Elevational positions used to reconstruct one volume are highlighted as green squares. (a) 1000-Hz PRF, the volume rate is 500 Hz, and the number of sampled elevational positions is 3. (b) 1125-Hz PRF, the volume rate is 125 Hz, and the number of sampled elevational positions is 9. (c) 1062.5-Hz PRF, the volume rate is 62.5 Hz, and the number of sampled elevational positions is 17.

There are two ways to implement the tradeoff between lower volume rate and better image quality (elevational–axial plane): the first method is to decrease the tilting frequency of the microfabricated mirror. One may have to increase the voltage of the driving signal to alleviate the decrease of tilting angle range due to using a frequency that is off the resonant frequency of the mirror. The second method is to change the PRF to make the ratio of the PRF to the mirror tilting frequency a noninteger number. As shown in (5) and (6), this effectively lowers the imaging volume rate and increases the number of elevational sampling positions, which translates to a better imaging quality. Fig. 20 shows three different PRFs (1000, 1125, and 1062.5 Hz) with the same mirror tilting frequency of 250 Hz. Through changing the PRF and corresponding ratio of the PRF to the mirror tilting frequency, there are three different combinations of volume rate and the number of sampled elevational positions. For example, if PRF = 1062.5 Hz and mirror tilting frequency is *F*_*m*_ = 250 Hz, then (PRF/*F*_*m*_) = 4.25, which belongs to condition 2 in (5) with *m* = 4. Therefore, the volume rate *F*_*v*_ = (*F*_*m*_/*m*) = 62.5 Hz, and the number of elevational sampling positions is *N*_*p*_ = (PRF/*F*_*v*_) = 17 based on (6).

The 2-D transducers could be configured to emit plane waves in azimuth and steer the beam along the elevational dimension, such as the FASTER approach. However, in practice, one may need to use a low channel count 2-D matrix array to achieve this; otherwise, a large number of elements may be involved to transmit a plane wave while effectively steering the beam in the elevational dimension. For a low channel count 2-D matrix array, e.g., the 32 × 32 (1024 elements) matrix array (Verasonics Inc., Kirkland, WA), it is feasible to achieve a similar 3-D imaging scheme, such as FASTER with a 256-channel ultrasound system (e.g., Verasonics Vantage 256). However, the size of the FOV is limited due to the small aperture size of the transducer.

One limitation of this study is that we did not introduce a method that would allow free-hand scanning with the ultrasound transducer and the microfabricated mirror without the water tank. The current FASTER implementation is limited to *in vitro* experiments underwater. Ongoing studies are being conducted to develop a device that would allow the microfabricated mirror to be attached to the ultrasound transducer for *in vivo* imaging.

## V. Conclusion

This article introduces a novel high volume rate 3-D ultrasound imaging technique based on a fast-tilting, water-immersible microfabricated mirror and conventional 1-D ultrasound transducers. Our technique, termed FASTER, takes advantage of the ultrafast imaging frame rate of plane waves and uses the microfabricated mirror to rapidly sweep the plane waves in the elevational dimension to realize high volume rate 3-D imaging. To the best of our knowledge, this is the first time that a water-immersible microfabricated mirror is applied to ultrafast plane wave imaging to realize 3-D ultrasound imaging. We demonstrated a 500-Hz imaging volume rate with accurate 3-D imaging reconstructions on a wire phantom and a tissue-mimicking phantom. Future work is geared toward developing a device that would allow free-hand scanning with the microfabricated mirror attached to the ultrasound transducer, as well as implementations of FASTER on various ultrasound techniques for 3-D blood flow imaging, 3-D shear wave elastography, 3-D functional ultrasound, and 3-D super-resolution ultrasound localization microscopy.

## Figures and Tables

**TABLE I:** Main Design Parameters of Water-Immersible Microfabricated Scanning Mirror

Parameters	Value
Hinges size (*l × w × h*) (*mm*^3^)	1.1 *×* 0.6 *×* 0.05
Mirror plate (*l × w × h*) (*mm*^3^)	20 *×* 3 *×* 0.2
Magnets size (*mm*^2^)	1.6 diameter *×* 0.8 height
Electromagnet size (*mm*^2^)	10 diameter *×* 25 height
Acrylic pieces size (*l × w × h*) (*mm*^3^)	9.5 *×* 11 *×* 0.8

**TABLE II:** Imaging and FASTER Configuration

Parameters	Value
Transmit frequency (MHz)	15.625
Elements	128
Lateral range (mm)	12.8
PRF(Hz)	20000
Imaging range (mm)	28.6
micro-fabricated mirror tilting frequency (HZ)	250
micro-fabricated mirror driving voltage (Vpp)	5 and 10
micro-fabricated mirror tilting angle	+7.5/−6.9 at 5 Vpp
range (°)	+12.6/−11.3 at 10 Vpp
micro-fabricated mirror tilting angle offset (*γ* in Eq. (3)) (°)	0
Distance from the transducer to the mirror (mm)	2.1
Volume rate (Hz)	500
Mechanical scanning step size (mm)	0.5

**TABLE III:** Contrast Values for Two Cysts at Different Depths Imaged by Mechanical Translation-Based 3-D Scanning and FASTER (Two Elevational–Axial Slices Were Used to Calculate the Values)

The center of cyst in axial dimension (mm)	Mechanical translation-based 3D scanning		FASTER 3D Imaging at 5 V		FASTER 3D Imaging at 10 V	
	Contrast (dB)	CNR	Contrast (dB)	CNR	Contrast (dB)	CNR
17	18.1	3.5	17.1	2.2	18.0	2.0
23	11.0	2.3	12.7	1.7	13.1	1.6
